# Oscillatory dynamics in paclitaxel-proteinoid networks

**DOI:** 10.1007/s44345-026-00047-x

**Published:** 2026-02-22

**Authors:** Panagiotis Mougkogiannis, Andrew Adamatzky

**Affiliations:** Unconventional Computing Laboratory, University of the West of England, Bristol, UK

**Keywords:** Paclitaxel, Proteinoids, Microtubules, Electrical oscillations, Cyclic voltammetry, Electrochemical impedance spectroscopy, Biomimetic systems

## Abstract

Paclitaxel is a widely used microtubule-targeting chemotherapeutic, yet its intrinsic electrochemical behavior remains poorly understood. Here we investigate the electrochemical and oscillatory properties of paclitaxel incorporated into proteinoid microspheres that mimic cellular environments. Using scanning electron microscopy, cyclic voltammetry, electrochemical impedance spectroscopy, and square-wave voltammetry, we compare pure paclitaxel, proteinoid–paclitaxel mixtures, and related proteinoid systems. Incorporation of paclitaxel induces the formation of interconnected fibrous networks and enhances electrical conductivity by nearly two orders of magnitude relative to pure paclitaxel. The proteinoid–paclitaxel system exhibits diffusion-controlled redox behavior, long-term stable electrical oscillations, and high signal coherence. Spectral and nonlinear analyses reveal distinct dynamical regimes, including chaotic behavior in mixed proteinoid systems. These results establish proteinoid–paclitaxel assemblies as electrically active biomimetic platforms and suggest their potential as model systems for studying microtubule-related bioelectrical phenomena and bio-inspired signal processing.

## Introduction

Microtubule dynamics play a key role in how cells work. Many chemotherapy drugs focus on these dynamics [1–5]. Knowing how these drugs work with microtubules is key for better cancer treatments [6]. Electrical oscillations are key in biological systems [7–11]. They help with neuronal signaling, cellular communication, and syncing cellular activities. In neurons, gamma oscillations (30–90 Hz) are tied to attention and memory. Lower frequencies, such as alpha and theta, relate to relaxation and sleep. Microtubules are important parts of the cytoskeleton [12]. They may help with bioelectrical activities. They could act as pathways for electrical signals to move inside cells. Understanding how MTs work with electricity helps us see their role in cell signaling, especially with drugs like paclitaxel. This knowledge is important for understanding diseases like cancer and neurodegeneration [13, 14].

**Fig. 1:**
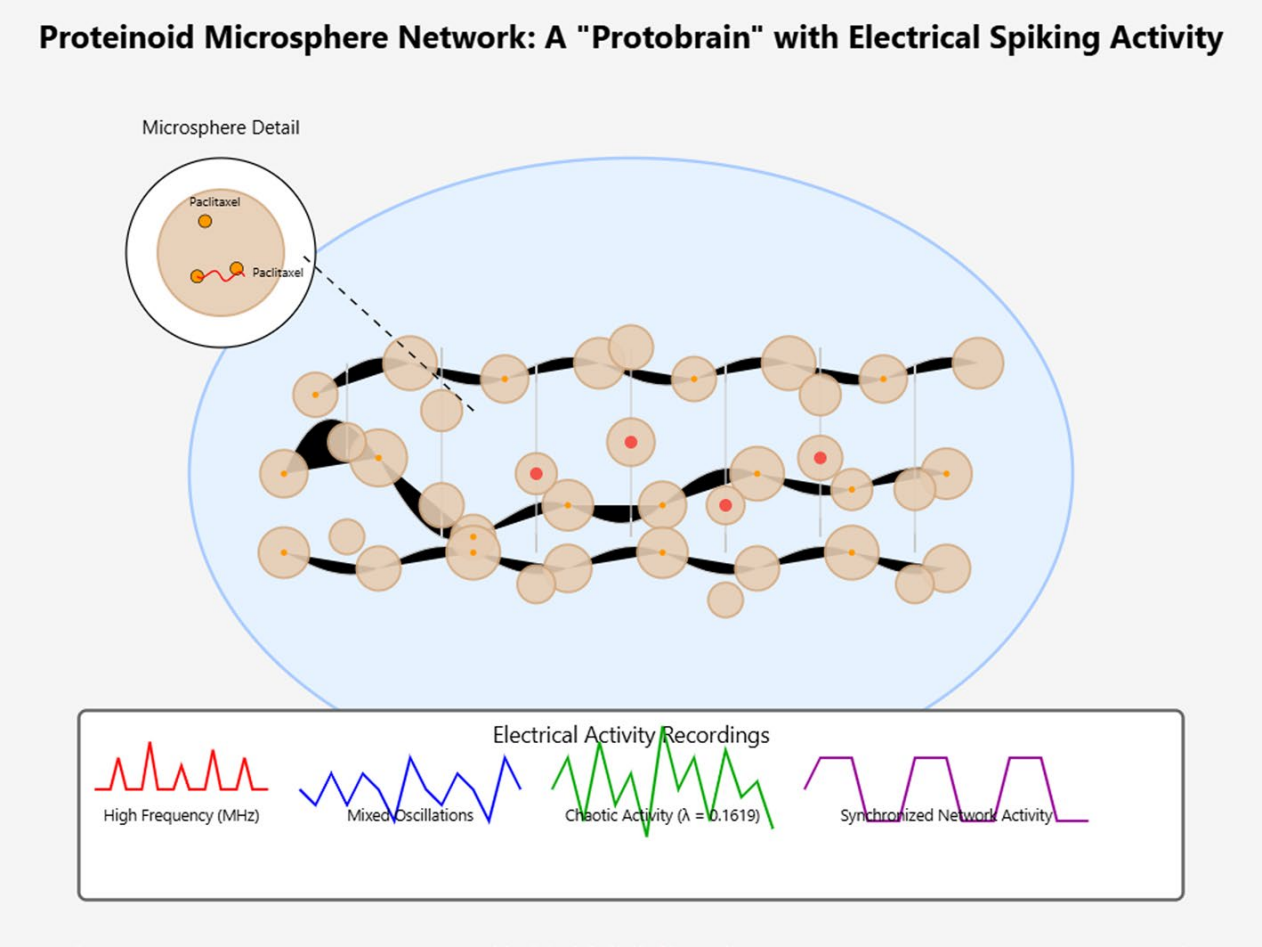
Proteinoid microsphere network as a ’protobrain’ exhibiting electrical spiking activity. The network of proteinoid-paclitaxel microspheres creates a neural-like structure. This structure can generate various electrical oscillation patterns. Electrical recordings reveal high-frequency spikes in the MHz range. They also show mixed oscillations, chaotic activity ($\lambda$ = 0.1619), and synchronized network activity. Inset: Detailed view of a single microsphere with paclitaxel binding sites and associated electrical signals. This model shows how adding paclitaxel to proteinoid structures creates complex bioelectrical activity. This activity may resemble the workings of neuronal networks linked to consciousness

Proteinoids are structures made by heating amino acids. They have interesting electrical properties and resemble some features of living systems. This suggests they could be a new way to study how drugs interact with microtubules [15]. Using paclitaxel, a known drug that stabilizes microtubules, in proteinoid microspheres may offer a unique method to study its effects on electrical activity. This could show how microtubule dynamics change in a limited space [16, 17]. Proteinoids show dynamic electrical activity. They have self-propagating currents and oscillations. This makes them a great way to study how paclitaxel affects these bioelectrical phenomena [15, 18]. Studying the frequency, strength, and patterns of electrical spikes in paclitaxel-embedded proteinoids shows how the drug works. It also helps us understand its impact on microtubule behavior (Fig. 1) [15, 18].

Proteinoids are synthetic, protein-like structures [19, 20] that provide a simplified biomimetic platform for studying bioelectrical phenomena and drug interactions [21, 22]. Their reduced cellular complexity enables isolation of specific processes, such as signal propagation, without interference from other cellular activities. This reductionist environment is particularly useful for probing how microtubule-stabilizing drugs influence electrical dynamics. By embedding paclitaxel within proteinoids, this study establishes a controllable model to investigate the coupling between microtubule dynamics and electrical oscillations, with potential applications in mechanistic studies and drug screening.

Recent studies reveal that isolated brain microtubules (MTs) show intrinsic electrical oscillations. They function as ionic transistors, generating, transmitting, and amplifying electrical signals [23–27]. These oscillations happen at frequencies of 43–47 Hz and around 90 Hz in non-stabilized MTs. Paclitaxel changes this behavior, fixing the oscillation to a main frequency of about 39 Hz. This electrical activity, driven by ion-permeable nanopores in the MT structure, suggests that MTs play a critical role in bioelectrical signaling. Adding paclitaxel to proteinoid systems can help us study how drugs impact MT dynamics and electrical activity. These systems have self-propagating currents and oscillations, making them useful for research in a biomimetic setting.

Paclitaxel affects microtubule dynamics. It causes microtubules to bundle and limits their spread in cells, even at low nanomolar levels. It also boosts microtubule stability [28]. Changes in microtubule organization and evidence show that proteinoids affect electrical signaling with some chemicals [15]. So, adding paclitaxel to proteinoids could lead to noticeable changes in their electrical activity [18]. The hypothesis is that paclitaxel changes microtubule dynamics. This may affect the proteinoid’s electrical oscillations. These changes might offer a real-time and sensitive way to measure how the drug interacts with its target. The idea of using proteinoids as a biomaterial with which to explore neurological consequences is not new [18]. Omeprazole is a proton pump inhibitor. It changes ion movement and membrane potential in proteinoid systems. This leads to different spike amplitudes and distributions [18]. Also, using Boolean logic with proteinoid-omeprazole systems has shown complex patterns in spiking data [18]. To assess this method, we can create paclitaxel-loaded proteinoids. We use techniques like electrospinning. This technique makes micro- and nanofibers that provide sustained drug delivery [29]. Characterizing paclitaxel-embedded proteinoids is key. We use dynamic light scattering and scanning electron microscopy. These techniques help confirm drug encapsulation and check the microspheres’ structural integrity. Researchers can track electrical activity changes with microelectrode arrays or voltage-sensitive dyes. This helps them link the drug’s presence to specific changes in the proteinoid’s electrical signaling [18]. Computational modeling can enhance experimental observations. It simulates how paclitaxel interacts with microtubules and the proteinoid matrix. This system could improve by adding fluorescently labeled tubulin. This change would let us see microtubule dynamics directly within the proteinoid. Combining paclitaxel and cisplatin can affect cell apoptosis levels [30–32]. This varies with the administration schedules. It may lead to different drug effects based on the cell traits of the surrounding population.

Studying electrical oscillations in synthetic systems such as proteinoids is challenging due to fabrication variability and the difficulty of detecting subtle electrical changes. Here, we address these limitations using standardized proteinoid preparation and microelectrode-based measurements, enabling reliable and reproducible detection of oscillatory behavior. This approach strengthens the robustness of our results and improves insight into microtubule-related electrical dynamics.

**Fig. 2:**
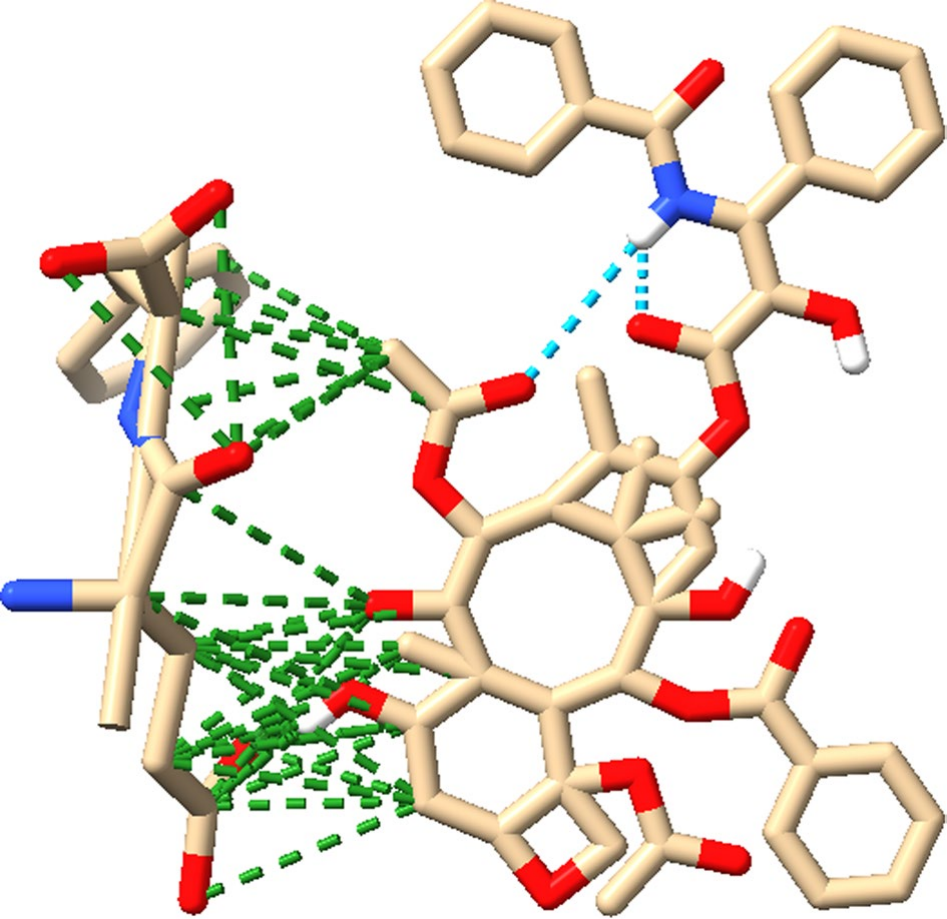
Molecular visualization of the Glu-Phe proteinoid (L-Glu:L-Phe) and Taxol complex using ChimeraX [39]. This figure shows key interactions that stabilize the system. The proteinoid structure is depicted with carbon atoms in beige, oxygen in red, and nitrogen in blue, while Taxol is integrated within the proteinoid matrix. Cyan dots show hydrogen bonds. They highlight polar interactions between Taxol and the proteinoid’s functional groups. These interactions likely help with structural stability and boost electrochemical activity seen in SWV experiments (Fig. 19). Green dots show hydrophobic contacts. These occur mainly between Taxol’s aromatic rings and the non-polar parts of the proteinoid. This interaction helps form connected fibrous networks, as noted in the study. These interactions lead to a 100-fold increase in electrical conductivity of the Proteinoid-Taxol Mixture over Pure Taxol. This boost allows for stable oscillatory behavior with a high signal coherence of 0.975. It also results in chaotic dynamics, shown by a Lyapunov exponent of $\lambda = 0.1619$. The visualized interactions show that Glu-Phe proteinoid boosts Taxol’s binding to microtubules. This might mimic the microtubule-driven MHz signal bursts seen in anesthetized patients [40]. It offers a synthetic model to study bioelectrical phenomena and consciousness mechanisms in synthetic biology

Microtubules (MTs) are central to cancer biology and are also critically involved in neurodegenerative disorders, where impaired MT stability and transport contribute to neuronal dysfunction; drugs such as paclitaxel may further modulate MT electrical oscillations, potentially affecting neuronal signaling and synaptic function [14, 33–36]. Squalenoyl-based nanoassemblies exhibit strong biocompatibility and self-assembly, making them attractive platforms for drug delivery and interaction studies [37], while growing interest in nanotherapeutics capable of crossing the blood–brain barrier further motivates this approach [38]. Accordingly, paclitaxel-loaded proteinoids provide a controllable model for probing MT dynamics and drug–MT interactions, with the Glu–Phe proteinoid–Taxol molecular structure (Fig. 2) explaining the enhanced electrochemical activity observed. Beyond cancer therapy, studying paclitaxel in proteinoid microspheres links molecular MT targeting to emergent bioelectrical dynamics, offering new insight into electrical signaling in synthetic and biological systems and suggesting potential relevance for neurological disorders involving coupled MT and electrical dysfunction.

## Experimental

Glu-Phe proteinoids are made using the thermal copolymerization method from Fox and Harada et. al [41, 42]. This process heats amino acids in dry conditions to create protein-like polymers. A mix of L-glutamic acid and L-phenylalanine was made in a 2:1 ratio. This ensures there is more dicarboxylic amino acid (glutamic acid) present, which is important for effective copolymerization [41]. Specifically, 10 g of L-glutamic acid was heated at 175–$180^\circ$C in a dry state until molten (approximately 30 minutes), during which it largely converts to its lactam form. Next, 5 g of L-phenylalanine went into the melted glutamic acid. The mix stayed at $170^\circ$C for 6 more hours under reflux to reduce oxidative damage. The reaction mixture cooled down. Then, it was dissolved in 50 ml of water. After that, we dialyzed it against distilled water for 48 hours. This step separated the insoluble proteinoid fraction (solid proteinoid) from the soluble fraction. The insoluble Glu-Phe proteinoid was lyophilized. This process produced about 2.5 g of solid proteinoid.

To make the Proteinoid-Taxol mixture for electrochemical tests, first we diluted 5 mg of Taxol from Sigma-Aldrich in 80 ml of distilled water. This gives a 0.0625 mg/ml Taxol solution. We stirred gently at room temperature to ensure it dissolves completely. The Glu-Phe proteinoid was dissolved in distilled water. It had a concentration of 0.0625 mg/ml, which matched the Taxol concentration. This made 80 ml of proteinoid solution, using 5 mg of proteinoid in 80 ml of water. We prepared the Proteinoid-Taxol mixture by mixing the two solutions in equal parts. This gave us 160 ml of a mixture with 2.5 mg of Taxol and 2.5 mg of Glu-Phe proteinoid, which is 0.03125 mg/ml for each.

**Fig. 3:**
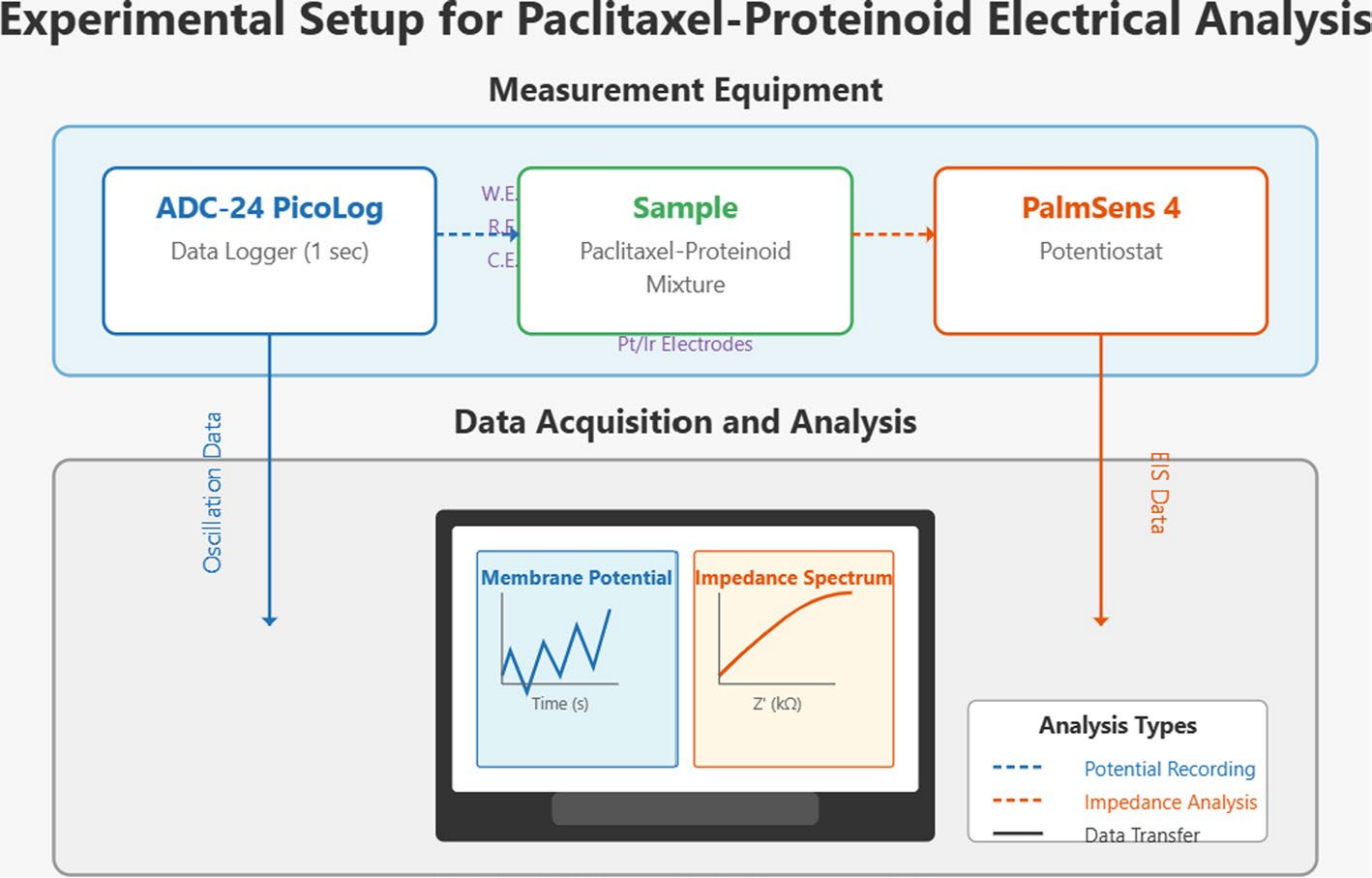
Schematic representation of the experimental setup for paclitaxel-proteinoid electrical analysis. The measurement system includes an ADC-24 PicoLog data logger (left) that records data at a 1 Hz sampling rate. It also uses a paclitaxel-proteinoid mixture sample with Pt/Ir electrodes in a three-electrode setup: the working electrode (*WE*), reference electrode (*RE*), and counter electrode (*CE*). It also includes a PalmSens 4 potentiostat (on the right) for electrochemical impedance spectroscopy (EIS). Data from both instruments goes to the computer system. This gives us two types of analyses: membrane potential oscillations in the time domain and impedance spectra in the frequency domain. Dashed lines show measurement pathways: blue for potential recording and orange for impedance analysis. Solid lines mean data transfer. The measurements in the microsecond range were performed using a Pico Technology 4824 A PicoScope 4000 A Series, a 20 MHz PC-based oscilloscope with 8 analogue channels, UKAS calibrated

We analyzed Pure Taxol and proteinoids, including Glu-Phe:Taxol Mixture and Glu:Phe Proteinoid. We used a few electrochemical methods: Square Wave Voltammetry (SWV), Galvanostatic Impedance Spectroscopy (EIS), and Cyclic Voltammetry (CV). We used a PalmSens4 potentiostat for these experiments (Fig. 3). This device is compact and portable, making it great for precise electrochemical measurements. The PalmSens4 has a three-electrode system. It includes platinum/iridium (Pt/Ir) working and counter electrodes. There’s also a reference electrode. The reference electrode was a Medium Serpentine Electrode Chip (MS100), product number MS100-DIE-1EA. This setup ensures accurate control over potential and current measurement during experiments. We chose Pt/Ir electrodes because they have great conductivity and chemical stability. This makes them ideal for studying the redox behavior of Taxol and proteinoid systems in water. For SWV, the PalmSens4 was configured with an equilibration time of $t_{\text {equilibration}} = {0}\,\text {s}$, a potential range from $E_{\text {begin}} = {-1.0}\,\text {V}$ to $E_{\text {end}} = {1.0}\,\text {V}$, a step potential of $E_{\text {step}} = {0.0001}\,\text {V}$, an amplitude of ${0.025}\,\text {V}$, and a frequency between 2 Hz to 50Hz. This setup enabled the detection of redox peaks, providing insights into the electron transfer processes involving Taxol and proteinoids. EIS measurements were conducted with an equilibration time of $t_{\text {equilibration}} = {10}\,\text {s}$, an applied current range of ${10}\,\text {mA}$, a DC current of $i_{\text {dc}} = {0.0}\,\upmu \text {A}$, an AC current amplitude of $i_{\text {ac}} = {0.2}\,\upmu \text {A}$, and a frequency scan across 77 frequencies from 9.9 Hz to 1.0 kHz. This setup made it possible to analyze interfacial properties. This includes charge transfer resistance at the electrode surface. CV experiments used the PalmSens4 potentiostat. The equilibration time was $t_{\text {equilibration}} = {10}\,\text {s}$. The potential swept from $E_{\text {begin}} = {0.0}\,\text {V}$ to $E_{\text {vertex1}} = {-1.0}\,\text {V}$, then to $E_{\text {vertex2}} = {1.0}\,\text {V}$. The step potential was $E_{\text {step}} = {0.1}\,\text {V}$ and the scan rate was ${0.1}\text {V}\,\text {s}^-{1}$. A total of 100 scans were performed. This setup helped us assess how well the Taxol-proteinoid systems can reverse redox reactions and transfer electrons. The Pt/Ir electrodes provided stable and repeatable measurements for all techniques. They reduced electrode wear during potential sweeps and frequency scans. We recorded electrophysiological data, like the membrane potential oscillations discussed earlier, using an ADC-24 PicoLog data logger. The ADC-24 PicoLog is a high-resolution data acquisition system capable of capturing multi-channel voltage signals with precision. It was configured with a sample rate of ${1}\,\text {s}$, meaning one data point was recorded every second, resulting in a sampling frequency of $f_{\text {sample}} = {1}\,\text {Hz}$. The sample rate was good enough to capture the slow movements of the compounds. They had mean periods ranging from hundreds to thousands of seconds. For example, Pure Taxol had an average time of $\overline{T} = {1490.98}\,\text {s}$, as shown in Table 5. The PalmSens4 potentiostat, paired with Pt/Ir electrodes and the ADC-24 PicoLog data logger, created a strong system. This setup is ideal for electrochemical and electrophysiological measurements. The electrochemical experiments showed the redox and interfacial properties of Taxol and proteinoids. The data logger provided accurate timing for the membrane potential changes. These tools allowed a thorough analysis of the Taxol-proteinoid systems. They connected the electrochemical behavior with potential consciousness-like dynamics in synthetic systems.

## Results and discussion

### Morphological analysis of pure paclitaxel and proteinoid/paclitaxel mixtures via scanning electron microscopy

**Fig. 4:**
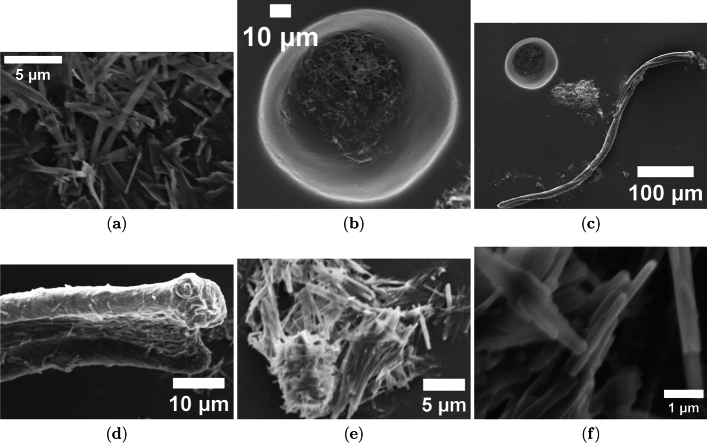
Scanning Electron Microscopy (SEM) Analysis of Pure Paclitaxel (Taxol) Morphology. (**a**) Needle-like crystals of pure paclitaxel appear elongated, measuring about 1–2 $\mu$m wide. This shape shows that crystals grew during sample preparation (scale bar: 5 $\mu$m). (**b**) Paclitaxel forms amorphous spherical clumps about 30 $\mu$m wide when the solvent evaporates (scale bar: 10 $\mu$m). (**c**) Larger paclitaxel aggregates show irregular surfaces. This may be due to recrystallization. These features were seen at a macroscale (scale bar: 100 $\mu$m). (**d**) Elongated paclitaxel crystals or aggregates have a tubular shape and a diameter of about 2 $\mu$m. This may be due to solvent effects (scale bar: 10 $\mu$m). (**e**) Dense network of paclitaxel crystals shows a fibrous texture. This matches the characteristics of crystalline paclitaxel. The scale bar is 5 $\mu$m. (**f**) High-magnification view shows paclitaxel crystals. You can see the tiny details of crystal facets at the nanoscale. The scale bar is 1 $\mu$m. These images show the diverse shapes of pure paclitaxel using SEM. They reflect how it crystallizes and clumps together based on different preparation methods

We studied the morphology of pure paclitaxel (Taxol Equivalent) and a 50/50 mixture of proteinoids with paclitaxel. We used scanning electron microscopy (SEM) to observe how their structures may create electrical oscillations. Figure 4 presents the SEM analysis of pure paclitaxel, revealing its intrinsic crystalline and aggregation behavior. In Fig. 4(a), paclitaxel forms needle-like crystals that are 1–2 $\mu$m wide. This shows crystal growth happening as the solvent evaporates. This is common for small organic molecules, like paclitaxel (MW 854 g/mol). Larger spherical aggregates, about 30 $\mu$m wide, are seen in Figure 4(b). These may form from solvent effects during sample preparation. At a larger scale (Fig. 4(c)), paclitaxel aggregates show uneven surfaces. This suggests that recrystallization is happening. A tubular morphology with a diameter of 2 $\mu$m is seen in Fig. 4(d), possibly an artifact of solvent-induced aggregation. The fibrous texture of paclitaxel crystals in Fig. 4(e) and the tiny crystal facets in Fig. 4(f) show its crystalline nature. This aligns its chemical properties as a diterpenoid.

**Fig. 5:**
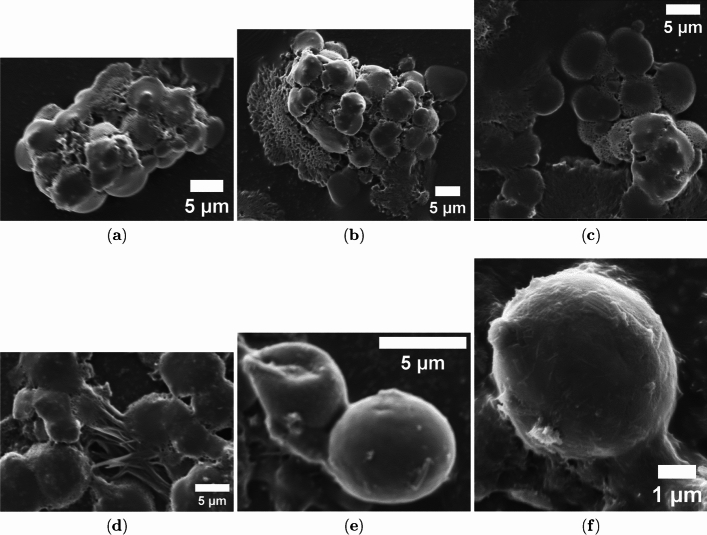
Scanning Electron Microscopy (SEM) Analysis of Proteinoid/Paclitaxel (Taxol) Mixture (50/50 vol/vol). (**a**) A cluster of proteinoid microspheres contains paclitaxel. These microspheres have irregular shapes and diameters between 2 and 5 $\mu$m, showing that the drug caused them to clump together (scale bar: 5 $\mu$m). (**b**) Dense cluster of proteinoid microspheres with paclitaxel shows a rough surface. This texture hints at paclitaxel incorporation (scale bar: 5 $\mu$m). (**c**) A larger group of proteinoid microspheres can be seen. They have diameters up to 5 $\mu$m. The mixture shows a uniform distribution of paclitaxel. (Scale bar: 5 $\mu$m). (**d**) An interconnected network of proteinoid microspheres may form a fibrous matrix. These microspheres, affected by paclitaxel’s stabilizing effects, have diameters of about 2–3 $\mu$m (scale bar: 5 $\mu$m). (**e**) Pair of proteinoid microspheres with smooth surfaces, showing paclitaxel encapsulation and a diameter of 3 $\mu$m (scale bar: 5 $\mu$m). (**f**) This high-magnification image shows one proteinoid microsphere. You can see tiny surface details, and it has a diameter of about 1 $\mu$m. The 50/50 vol/vol mixture of proteinoid and paclitaxel affects its structure. (Scale bar: 1 $\mu$m). These images show the shape of proteinoid structures changed by paclitaxel. This helps us study their electrical oscillation in a biomimetic system

The 50/50 vol/vol mixture of proteinoids and paclitaxel, seen in Fig. 5, shows a unique morphology. This morphology features proteinoid microspheres that are changed by the addition of paclitaxel. Figure 5(a) shows clusters of irregular proteinoid microspheres. These microspheres have diameters of 2–5 $\mu$m. This suggests that paclitaxel causes aggregation in the proteinoid matrix. The rough texture of these microspheres in Fig. 5(b) shows that paclitaxel is successfully encapsulated. This may affect the electrical properties of the proteinoid system. In Fig. 5(c), we see a larger cluster of microspheres with even paclitaxel distribution. Figure 5(d) displays a web of microspheres that creates a fibrous matrix. This may happen because paclitaxel stabilizes the assembly of proteinoids. The smooth surfaces of the paired microspheres in Fig. 5(e) and the fine details on a single microsphere in Fig. 5(f), which is about 1 $\mu$m wide, reveal how paclitaxel changes proteinoid shape.

The pronounced contrast between the needle-like crystals of pure paclitaxel and the rounded, interconnected proteinoid microspheres demonstrates that paclitaxel actively modifies proteinoid self-assembly, likely altering matrix properties such as surface charge and porosity and thereby influencing ion transport and electrical oscillations. The fibrous network observed in the mixture (Fig. 5(d)) is consistent with enhanced electrical conductivity and mirrors the microtubule networks stabilized by paclitaxel, as reported by Gutierrez et al. [23]. These structure–function relationships directly link the morphology of proteinoid–paclitaxel assemblies to their emergent electrical behavior and support their use as biomimetic models for studying microtubule dynamics.

The structures of pure paclitaxel and its mix with proteinoids align with larger studies. These studies focus on paclitaxel’s complex structure and how it acts in different systems. Paclitaxel’s structure comes from its biosynthesis and side chain variety. Enzymes that form these side chains can alter the final structure and activity of Taxol derivatives [43]. Production methods, such as extracting from Taxus plants or using microbes, affect physical properties and yield. Semi-synthesis strategies can create different crystalline or amorphous forms [44]. In drug delivery, paclitaxel forms spherical, amorphous structures in glycyrrhizic acid micelles. This improves oral bioavailability and differs from its crystalline form [45]. The total synthesis of paclitaxel shows the challenges of its complex ring structure and stereocenters. These features lead to its diverse forms under various conditions [46]. Analytical profiling shows that paclitaxel’s shape can change a lot. This depends on its formulation and environmental factors. It shows how pure paclitaxel’s crystal needles are different from the microspherical clumps in the proteinoid mixture [47]. These findings help explain the shape changes we saw in our SEM analysis. They suggest that paclitaxel’s structure and its interactions with proteinoids may be key in affecting the electrical oscillations we studied.

### Electrochemical characterization of paclitaxel via cyclic voltammetry

**Fig. 6:**
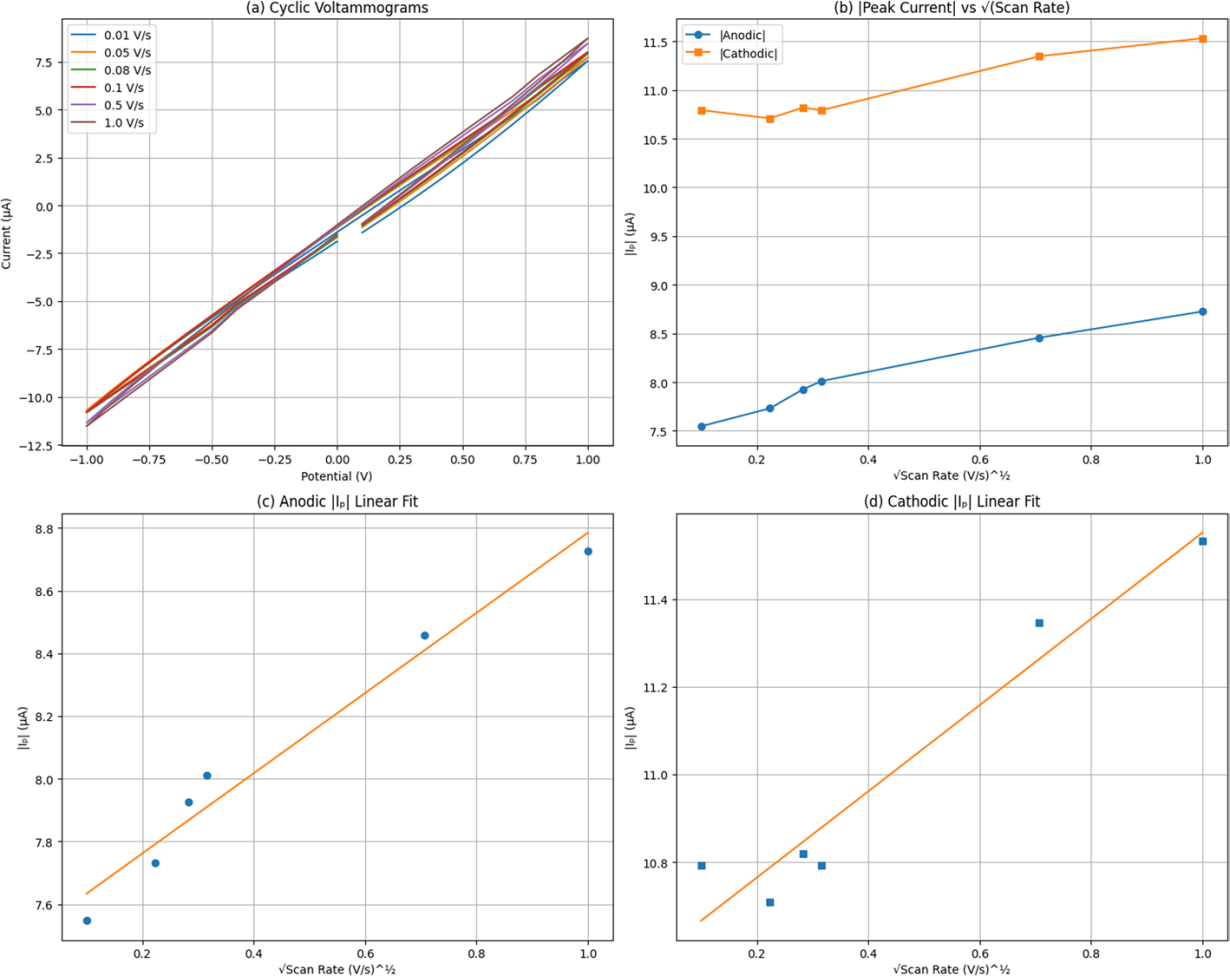
Electrochemical Analysis of Proteinoid CV Response. (**a**) Cyclic voltammograms recorded at scan rates of 0.01, 0.05, 0.08, 0.1, 0.5, and 1.0 V/s. (**b**) Absolute anodic and cathodic peak currents plotted against the square root of scan rate. (**c**) Linear fit of anodic peak current vs. $\sqrt{v}$. (**d**) Linear fit of cathodic peak current vs. $\sqrt{v}$. Peak currents increase steadily with scan rate, ranging from 7.547–8.725 $\mu$A (anodic) and 10.709–11.532 $\mu$A (cathodic), indicating ohmic behavior with limited diffusional contribution

We studied the electrochemical properties of pure paclitaxel (Taxol Equivalent) using cyclic voltammetry (CV) to understand its redox behavior and its role in the proteinoid/paclitaxel system. Cyclic voltammograms were recorded at scan rates from 0.01 V/s to 1.0 V/s over a potential range of −1 V to 1 V (Fig. 6a). Peak analysis in Table 1 shows that anodic peak potentials remain constant at 1000.278 mV and cathodic peak potentials at −1000.199 mV across all scan rates, indicating stable redox processes. The anodic peak current increases modestly from 7.547 $\mu$A to 8.725 $\mu$A (15.6% increase), while the cathodic peak current rises from 10.793 $\mu$A to 11.532 $\mu$A (6.8% increase) over a 100-fold scan rate range. Figure 6b displays the absolute values of peak currents versus the square root of scan rate. Linear regression analysis (Fig. 6c, d) yields $R^2 = 0.969$ for the anodic process and $R^2 = 0.982$ for the cathodic process, suggesting some degree of scan rate dependence. However, the modest current increase (only 15.6% over a 100-fold scan rate range) is significantly less than the 10-fold (1000%) increase expected for purely diffusion-controlled processes according to the Randles–Ševčík equation:

1 $$\begin{aligned} i_p = (2.69 \times 10^5) n^{3/2} A D^{1/2} C v^{1/2} \end{aligned}$$

where $i_p$ is the peak current (A), $n$ is the number of electrons transferred (assumed as 1), $A$ is the electrode area (cm$^2$), $D$ is the diffusion coefficient (cm$^2$/s), $C$ is the concentration (mol/cm$^3$) (1 mM), and $v$ is the scan rate (V/s).

Initial application of the Randles-Ševčík equation, using the slope from Fig. 6c and the geometric electrode area of a needle-like electrode (diameter 0.40 mm, exposed length 2 mm, $A_{\text {geom}} = 0.0251 \, \text {cm}^2$ for the lateral surface), yielded an apparent diffusion coefficient of $3.59 \times 10^{-8} \, \text {cm}^2/\text {s}$. For the Taxol-Proteinoid Mixture (discussed in subsequent sections), the calculated value was $1.73 \times 10^{-4} \, \text {cm}^2/\text {s}$. This latter value significantly exceeds the theoretical maximum for aqueous diffusion (H^+^: $D_{\text {max}} \approx 9 \times 10^{-5} \, \text {cm}^2/\text {s}$), revealing that the observed electrochemical behavior is not purely diffusion-controlled. The minimal scan rate dependence-only 15.6% current increase over a 100-fold scan rate range for Pure Taxol-indicates that double-layer charging and ohmic resistance dominate the electrochemical response rather than Faradaic electron transfer. True diffusion-controlled systems exhibit current scaling with $\sqrt{v}$, yielding a 10-fold increase ($\sqrt{100} = 10$) for a 100-fold scan rate variation. The observed meager hysteresis and slight curve tilts in the voltammograms are consistent with predominantly capacitive behavior at the proteinoid-electrode interface. The Randles-Ševčík equation strictly applies only when migration is negligible, typically achieved by adding excess supporting electrolyte (greater than 100-fold molar excess over analyte). Our proteinoid-based medium lacks conventional supporting electrolyte, allowing electric field-driven ion migration to contribute significantly alongside diffusion. This mixed transport mechanism invalidates the pure diffusion assumption and leads to anomalously high apparent diffusion coefficients. If we assume a physically reasonable diffusion coefficient for organic molecules ($D \approx 10^{-6} \, \text {cm}^2/\text {s}$), back-calculation suggests an effective electroactive area of $A_{\text {eff}} \approx 0.33 \, \text {cm}^2$, approximately 13-fold larger than the geometric area. This discrepancy may arise from surface roughness of the proteinoid film, formation of a porous electroactive layer, or three-dimensional diffusion through the hydrated proteinoid matrix. Rather than classical Faradaic redox chemistry, the Taxol-Proteinoid system exhibits mixed capacitive-Faradaic behavior with double-layer capacitance as the dominant contribution, ohmic resistance contributing to the near-linear voltammograms, mixed diffusion-migration due to absence of supporting electrolyte, and minor Faradaic contributions evident from slight hysteresis and stable peak potentials. While not conforming to classical diffusion-controlled redox kinetics, these properties are advantageous for applications in capacitive biosensing, bioelectrical signal transduction, and the stable electrical oscillations observed in subsequent sections.

**Fig. 7:**
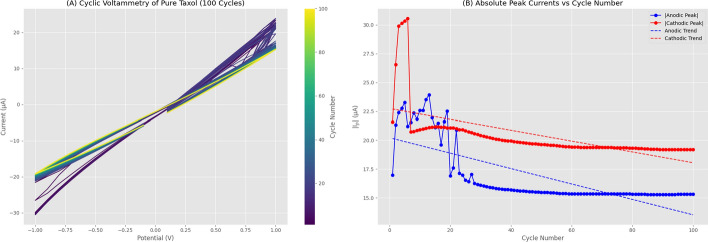
Electrochemical Stability of Pure Taxol Over 100 Consecutive Cycles. (**A**) Cyclic voltammograms for 100 cycles recorded at a scan rate of 0.1 V/s over a potential range of −1.0 to 1.0 V, color-coded by cycle number (purple = early cycles, yellow = late cycles). The nearly linear current-voltage relationship with minimal hysteresis demonstrates predominantly ohmic/capacitive behavior rather than Faradaic redox processes. The consistent voltammogram shape across all cycles confirms that the observed capacitive response is stable and not an artifact of electrode passivation, surface fouling, or degradation of the paclitaxel sample. (**B**) Absolute anodic peak currents ($|i_{p,a}|$, blue circles) and cathodic peak currents ($|i_{p,c}|$, red circles) extracted from each cycle and plotted versus cycle number. Linear regression analysis reveals a modest downward trend for anodic currents (slope = −0.0669 $\mu$A/cycle, intercept = 20.22 $\mu$A, $R^2 = 0.5586$) and a slight upward trend for cathodic currents (slope = 0.0469 $\mu$A/cycle, intercept = −22.74 $\mu$A, $R^2 = 0.3699$). Mean values stabilize at $16.84 \pm 2.59~\mu$A (anodic) and $-20.37 \pm 2.22~\mu$A (cathodic). The small drift in peak currents over 100 cycles (approximately 15% decrease for anodic, 7% increase for cathodic) indicates reasonable electrochemical stability of the predominantly capacitive response. The larger scatter in early cycles (1–20) likely reflects electrode conditioning, while the stabilization in later cycles (20–100) confirms reproducible capacitive charging/discharging behavior at the paclitaxel-electrode interface

We performed cyclic voltammetry on pure Taxol for 100 consecutive cycles to assess electrochemical stability. The results are shown in Fig. 7. Figure 7A displays overlaid CV curves color-coded by cycle number, revealing the evolution and stabilization of the voltammetric response. Figure 7B plots the absolute peak currents versus cycle number with linear regression fits. The mean peak anodic current was $16.84 \pm 2.59 \, \mu \text {A}$ and mean peak cathodic current was $-20.37 \pm 2.22 \, \mu \text {A}$. Linear regression shows a slight downward trend for anodic currents (slope = $-0.0669 \, \mu \text {A/cycle}$, $R^2 = 0.5586$) and a modest upward trend for cathodic currents (slope = $0.0469 \, \mu \text {A/cycle}$, $R^2 = 0.3699$), indicating reasonable electrochemical stability over 100 cycles despite the predominantly capacitive response.

**Table 1:** Peak analysis results from cyclic voltammetry of paclitaxel

Scan Rate (V/s)	Anodic Peak Current ($\mu$A)	Anodic Peak Potential (mV)	Cathodic Peak Current ($\mu$A)	Cathodic Peak Potential (mV)
0.01	7.547	1000.278	−10.793	−1000.199
0.05	7.731	1000.278	−10.710	−1000.199
0.08	7.926	1000.278	−10.820	−1000.199
0.10	8.011	1000.278	−10.793	−1000.199
0.50	8.457	1000.278	−11.346	−1000.199
1.00	8.725	1000.278	−11.532	−1000.199

This table reports the anodic and cathodic peak currents and potentials at scan rates from 0.01 to 1.0 V/s. Both anodic and cathodic peak potentials remain stable (1000.278 mV and –1000.199 mV, respectively), indicating consistent redox behaviour. Peak currents increase with scan rate, and show a linear dependence on the square root of the scan rate ($R^2 = 0.982$), suggesting diffusion-controlled kinetics

### Electrochemical impedance spectroscopy analysis of paclitaxel

**Fig. 8:**
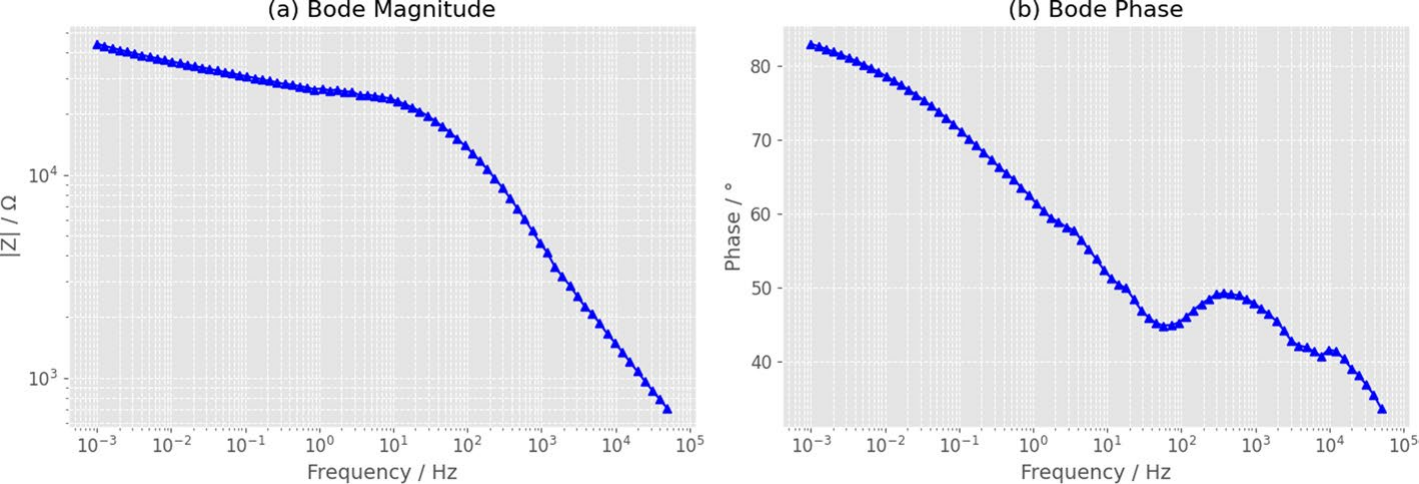
Electrochemical impedance spectroscopy of pure paclitaxel (Taxol). **(a)** Bode magnitude plot showing impedance $|Z|/\Omega$ as a function of frequency $f/\textrm{Hz}$ on logarithmic scales. The impedance drops from about $5 \times 10^4~\Omega$ at low frequencies ($10^{-3}~\textrm{Hz}$) to $7 \times 10^2~\Omega$ at high frequencies ($10^5~\textrm{Hz}$). There is a noticeable slope change between $10$ and $100~\textrm{Hz}$, indicating a shift in charge transfer mechanisms. **(b)** Bode phase plot demonstrating the phase angle $\phi /^{\circ }$ response across the frequency spectrum. The phase angle steadily decreases from $82^\circ$ at low frequencies to about $34^\circ$ at high frequencies. A notable transition occurs around $100{-}200~\textrm{Hz}$, where the phase angle reaches a local minimum of approximately $45^\circ$, then rises to a local maximum around $50^\circ$, before continuing its downward trend. This phase behavior indicates a plateau region, suggestive of a complex electrochemical interface with multiple time constants. Such behavior is consistent with a distributed relaxation process associated with Taxol’s interaction at the electrode surface

We analyzed the electrochemical impedance spectroscopy (EIS) of pure paclitaxel (Taxol) using Bode plots (Fig. 8) to characterize its frequency-dependent electrochemical behavior. Prior to impedance measurements, the system stability and causality requirements were carefully verified, as these are fundamental prerequisites for valid EIS analysis. Each sample was equilibrated for 600 s at open-circuit potential while continuously monitoring the potential drift. Only systems exhibiting drift rates below 2 mV/min were considered sufficiently stable for impedance measurements, ensuring that the observed impedance response resulted solely from the applied AC perturbation and not from spontaneous electrochemical processes or time-dependent changes in the electrode-electrolyte interface. The impedance magnitude ($|Z|/\Omega$) and phase angle ($\phi /^\circ$) were measured over a frequency range spanning seven decades, from $10^{-3}$ Hz to $10^{5}$ Hz, using galvanostatic mode with an AC current amplitude of 0.2 $\mu$A superimposed on a DC bias of 0 $\mu$A. The galvanostatic mode was selected rather than potentiostatic mode because it provides more stable measurements for high-impedance systems like paclitaxel, where the large electrode-electrolyte impedance could lead to distorted potentiostatic responses. Figure 8a shows that the impedance magnitude decreases steadily from approximately $5 \times 10^{4}\,\Omega$ at the lowest measured frequency ($10^{-3}$ Hz) to $7 \times 10^{2}\,\Omega$ at the highest frequency ($10^{5}$ Hz), spanning nearly two orders of magnitude across the measured frequency range. The frequency-dependent impedance behavior reveals distinct regions with different characteristic slopes on the log-log plot. At very low frequencies ($f < 1$ Hz), the impedance exhibits a steep negative slope of approximately $-1$ on the log-log scale, which is characteristic of capacitive behavior dominated by double-layer charging at the paclitaxel-electrode interface. A noticeable slope change occurs between 10 and 100 Hz, indicating a transition from capacitance-dominated response to mixed capacitive-resistive behavior where charge transfer processes begin to contribute significantly. At high frequencies ($f> 10^{3}$ Hz), the impedance magnitude approaches a nearly constant asymptotic value of approximately $7 \times 10^{2}\,\Omega$, which corresponds to the solution resistance ($R_s$) between the working and reference electrodes. This transition from capacitive to resistive dominance across the frequency spectrum is typical of electrochemical systems with complex interfacial properties and reflects the interplay between charge storage (capacitance) and charge transfer (resistance) processes at different timescales.

The phase angle response displayed in Fig. 8b provides complementary and essential information about the frequency-dependent electrochemical behavior that cannot be fully captured by impedance magnitude alone. The phase angle decreases progressively from $82^\circ$ at low frequencies ($10^{-3}$ Hz) to approximately $34^\circ$ at high frequencies ($10^{5}$ Hz), confirming the fundamental transition from capacitance-dominated response (where an ideal capacitor would exhibit $\phi = -90^\circ$) to resistance-dominated response (where an ideal resistor exhibits $\phi = 0^\circ$). The failure to reach the ideal capacitive limit of $-90^\circ$ at low frequencies indicates that the system does not behave as a perfect capacitor, even in the regime where capacitive effects are strongest. This deviation from ideal behavior suggests the presence of leakage currents, parallel resistive pathways, or distributed impedance characteristics that prevent purely capacitive response. Most notably, the phase angle exhibits complex non-monotonic behavior in the intermediate frequency region around $100\text {--}200$ Hz, where a local minimum of approximately $45^\circ$ is observed, followed by a rise to a local maximum around $50^\circ$, before resuming the downward trend toward lower phase angles at higher frequencies. This characteristic phase plateau or shoulder is a well-established signature of multiple time constants operating simultaneously in the electrochemical system. In the context of electrochemical impedance theory, such behavior indicates the presence of distributed relaxation processes rather than a single, well-defined relaxation time. For paclitaxel specifically, this distributed behavior likely originates from surface heterogeneity at the electrode-electrolyte interface, where the complex molecular architecture of paclitaxel creates multiple distinct microenvironments. The paclitaxel molecule contains numerous functional groups including hydroxyl groups, ester linkages, amide bonds, and aromatic rings, each of which can interact differently with the electrode surface through various mechanisms such as physisorption, chemisorption, hydrogen bonding, or $\pi$-$\pi$ stacking interactions. Each type of interaction contributes its own characteristic relaxation time, and the collective superposition of these relaxation processes manifests as the distributed phase angle response observed experimentally. The presence of multiple time constants also suggests that paclitaxel molecules may adopt different conformations or aggregation states at the electrode surface, with each configuration exhibiting distinct electrochemical response characteristics. This interpretation is consistent with the known behavior of paclitaxel in solution, where the molecule can exist in various conformational states due to rotational freedom around single bonds and intramolecular hydrogen bonding patterns. The observation of distributed impedance behavior has important implications for understanding how paclitaxel interacts with biological membranes and protein targets, as similar heterogeneity in binding modes may contribute to its pharmacological activity.

**Fig. 9:**
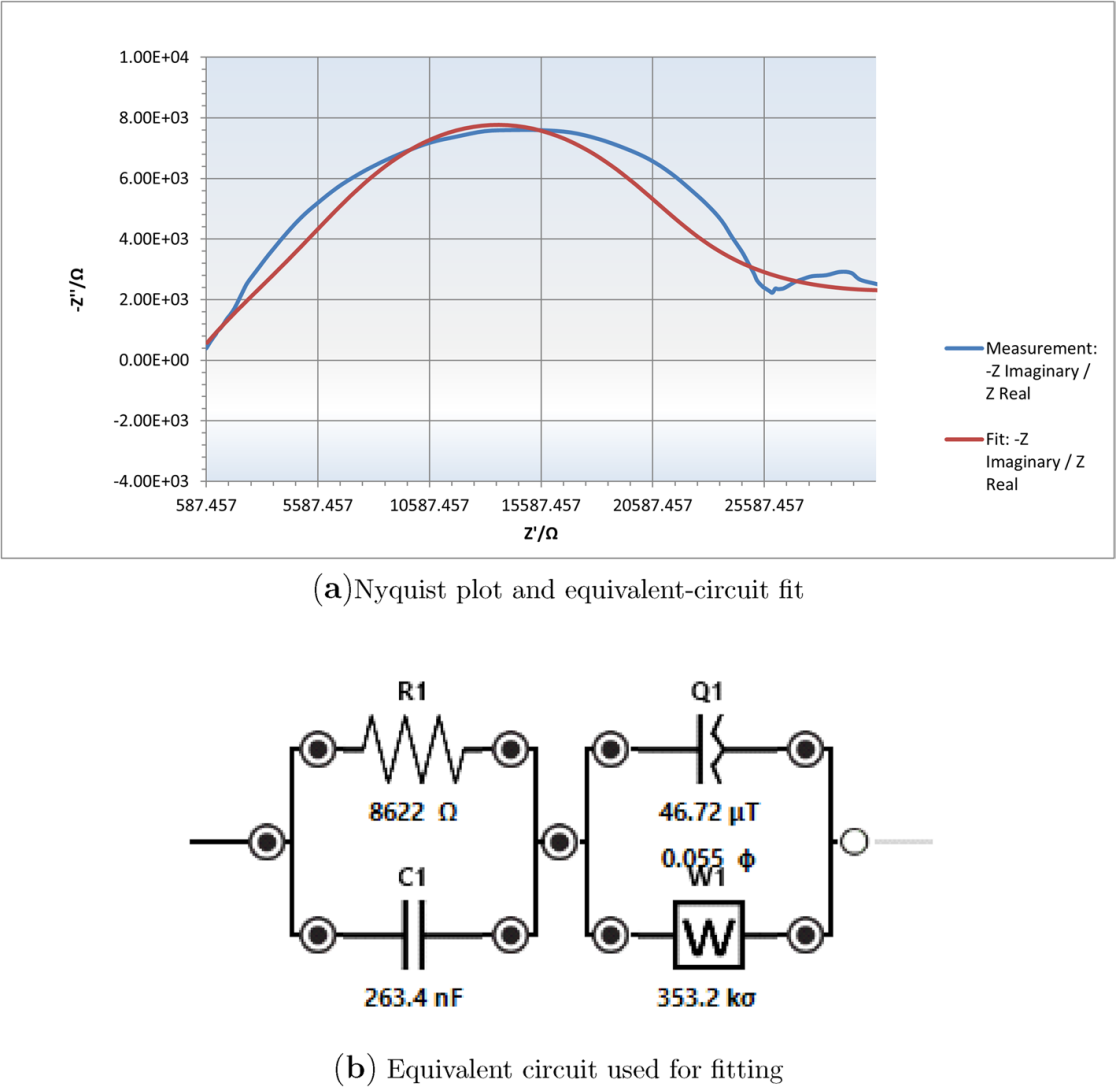
Equivalent-circuit fitting of galvanostatic impedance data using the $(RC)(QW)$ model. (a) Nyquist plot (blue) of the measured impedance response and the fitted $(RC)(QW)$ model (red). The circuit consists of a parallel resistor–capacitor pair $(R_{1} \parallel C_{1})$ in series with a constant-phase element $Q_{1}$ (with exponent $n_{1}$) and a semi-infinite Warburg diffusion element $W_{1}$. The fitted parameters are: $R_{1} = 8.62 \times 10^{3}\,\Omega$ (27.09% error), $C_{1} = 2.63 \times 10^{-7}\,\textrm{F}$ (29.10% error), $Q_{1} = 4.67 \times 10^{-5}\,\textrm{T}$ (13.88% error), $n_{1} = 0.055$ (31.19% error), and $W_{1} = 3.53 \times 10^{5}\,\sigma$ (22.79% error). (b) Schematic representation of the $(RC)(QW)$ equivalent circuit used for fitting, showing the arrangement of $R_{1}$, $C_{1}$, $Q_{1}$, and the Warburg element $W_{1}$. The low value of the CPE exponent $n_{1}$ reflects strong interfacial dispersion, while $W_{1}$ accounts for diffusion-limited behaviour at low frequencies. The goodness of fit is supported by a chi-squared value of $\chi ^{2} = 1.54\times 10^{-2}$, confirming that this equivalent circuit provides an adequate physical description of the impedance response

To quantitatively describe the impedance behavior and extract physically meaningful parameters, we performed equivalent circuit fitting on the Nyquist plot representation of the impedance data (Fig. 9a). Upon careful examination of the Nyquist plot, we observe a primary semicircular arc in the high-to-intermediate frequency region (left side of the plot), followed by a secondary feature at lower frequencies (right side of the plot) that extends toward the Warburg diffusion region. This two-feature structure indicates that at least two distinct electrochemical processes with different characteristic time constants are operative in the paclitaxel system. To properly account for this complexity, we employed a (*RC*)(*QW*) equivalent circuit model (Fig. 9b), which consists of a parallel resistor-capacitor pair ($R_1 \parallel C_1$) in series with a constant phase element ($Q_1$) and a semi-infinite Warburg diffusion element ($W_1$). The physical interpretation of each circuit element is as follows: $R_1$ represents the charge transfer resistance at the paclitaxel-electrode interface, quantifying the kinetic barrier for electron transfer between the electrode and redox-active species in solution or adsorbed on the surface. $C_1$ represents the double-layer capacitance arising from charge separation at the electrode-electrolyte interface, where ionic species in solution organize into a diffuse layer in response to the electrode’s surface charge. The constant phase element $Q_1$ accounts for non-ideal capacitive behavior arising from surface roughness, porosity, or heterogeneity in the electrode coating, with its exponent $n_1$ quantifying the degree of deviation from ideal capacitance (where $n_1 = 1$ corresponds to a pure capacitor and $n_1 = 0$ corresponds to a pure resistor). Finally, the Warburg element $W_1$ describes semi-infinite linear diffusion, accounting for mass transport limitations that become dominant at low frequencies where the AC perturbation period becomes long enough for concentration gradients to develop in the solution adjacent to the electrode. The fitted parameters obtained through non-linear least-squares optimization are: $R_1 = 8.62 \times 10^{3}\,\Omega$ with a relative fitting error of 27.09%, $C_1 = 2.63 \times 10^{-7}\,\textrm{F}$ (29.10% error), $Q_1 = 4.67 \times 10^{-5}\,\textrm{T}$ where T denotes the units of a CPE (13.88% error), $n_1 = 0.055$ (31.19% error), and $W_1 = 3.53 \times 10^{5}\,\sigma$ where $\sigma$ is the Warburg coefficient with units of $\Omega \,\text {s}^{-1/2}$ (22.79% error). The charge transfer resistance value of 8.62 k$\Omega$ is relatively large, indicating sluggish electron transfer kinetics, which is expected for paclitaxel given its large molecular weight (854 g/mol) and sterically hindered structure that may limit close approach to the electrode surface. The double-layer capacitance of 0.263 $\mu$F falls within the typical range for organic molecule-coated electrodes ($0.1\text {--}1\,\mu \text {F/cm}^2$ for our electrode area of approximately 0.025 cm$^2$), confirming reasonable physical consistency. Most significantly, the CPE exponent value of $n_1 \approx 0.055$ is extremely low, far from the ideal value of 1.0 that would indicate pure capacitive behavior. This very low exponent reflects profound interfacial dispersion and heterogeneity, consistent with a rough, porous, or non-uniform paclitaxel layer on the electrode surface. Such strong deviation from ideality suggests that the paclitaxel coating exhibits substantial variations in thickness, density, or composition across the electrode surface, creating a distribution of local capacitances rather than a uniform interfacial capacitance. The Warburg coefficient of $3.53 \times 10^{5}\,\Omega \,\text {s}^{-1/2}$ quantifies the diffusion-limited mass transport contribution, with larger values indicating more pronounced diffusion limitations. The overall goodness of fit is quantified by the chi-squared value of $\chi ^2 = 1.54 \times 10^{-2}$, which is acceptably small and confirms that the (*RC*)(*QW*) equivalent circuit provides a reasonable mathematical description of the measured impedance response. However, the relatively high fitting errors for individual parameters (ranging from 13.88% to 31.19%) indicate substantial uncertainty in the extracted values and suggest that this four-parameter model, while superior to the single-semicircle model, still represents a simplified approximation of the true electrochemical system. These fitting uncertainties likely arise from several sources: first, the overlap between the two semicircular features makes it difficult to determine where one process ends and the other begins; second, the limited number of frequency points (77 frequencies scanned) may be insufficient to fully resolve all features of the complex impedance spectrum; third, experimental noise and instrumental limitations introduce measurement uncertainty that propagates into parameter uncertainties; and fourth, the fundamental assumption that the system can be described by a discrete equivalent circuit with lumped elements may not fully capture the truly distributed nature of the paclitaxel-electrode interface.

### Electrochemical characterization of paclitaxel-proteinoid mixture via cyclic voltammetry

**Fig. 10:**
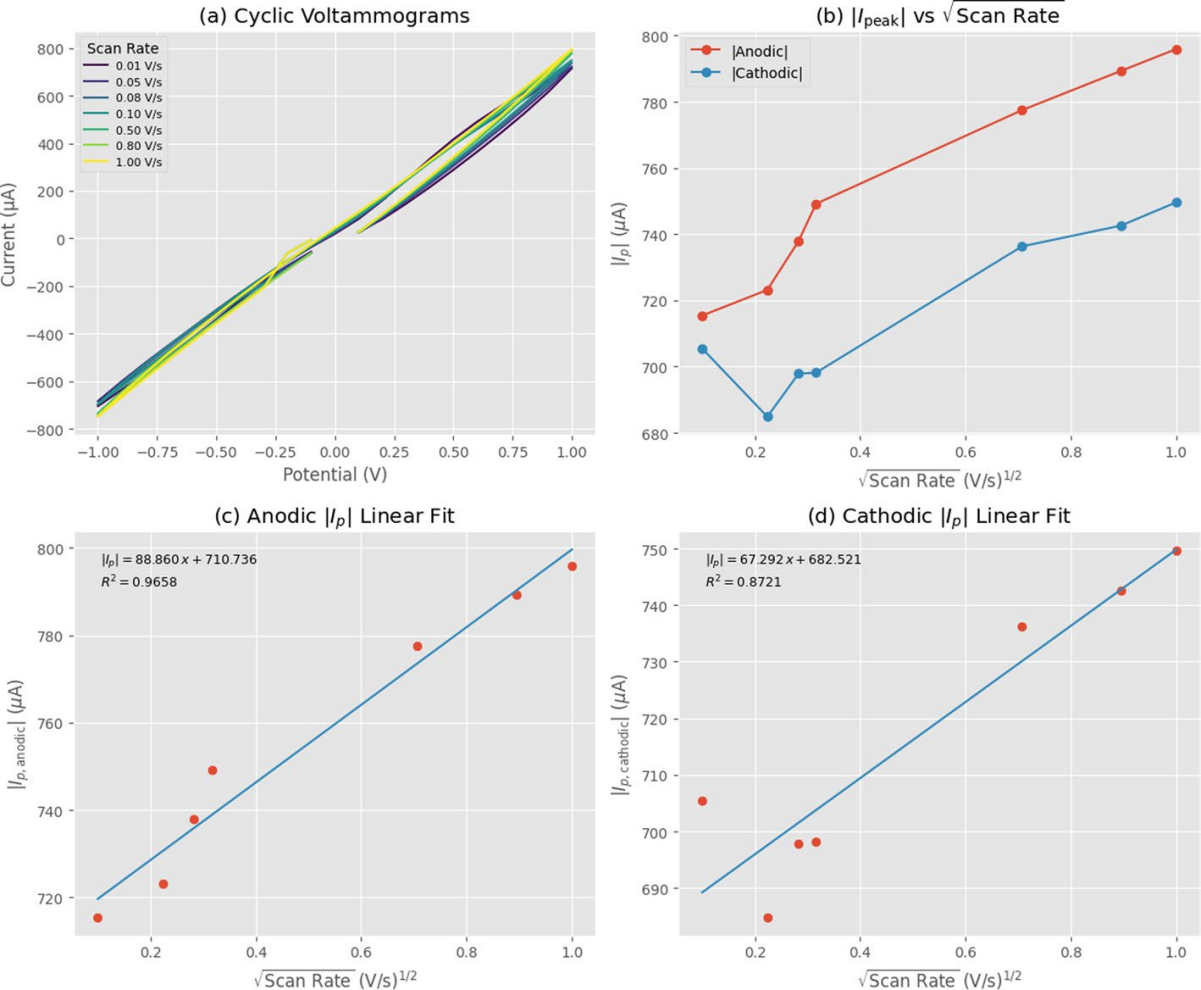
Electrochemical Analysis of Taxol-Proteinoid Mixture via Cyclic Voltammetry. (**a**) Cyclic voltammograms recorded at scan rates of 0.01, 0.05, 0.08, 0.10, 0.50, 0.80, and 1.00 V/s over a potential range of −1.0 to 1.0 V. The voltammograms exhibit near-linear current-voltage relationships with modest hysteresis, characteristic of mixed capacitive-Faradaic behavior. Unlike pure Taxol (Fig. 6), the Taxol-Proteinoid mixture shows substantially higher current magnitudes (reaching $\pm 800~\mu$A compared to $\pm 30~\mu$A for pure Taxol), indicating that the proteinoid matrix significantly enhances electrochemical activity. (**b**) Absolute peak currents ($|i_{p,a}|$, red circles for anodic; $|i_{p,c}|$, blue circles for cathodic) plotted versus the square root of scan rate ($\sqrt{v}$). Both anodic and cathodic currents increase with $\sqrt{v}$, demonstrating scan rate dependence. The anodic current rises from 715 $\mu$A at 0.01 V/s to 796 $\mu$A at 1.00 V/s (11.3% increase), while the cathodic current increases from 705 $\mu$A to 749 $\mu$A (6.2% increase). Although these increases are larger in absolute terms than pure Taxol, the relative changes remain modest (6–11% over a 100-fold scan rate range versus the 1000% expected for pure diffusion control). (**c**) Linear regression fit of absolute anodic peak current versus $\sqrt{v}$, yielding $|i_{p,a}| = 88.860x + 710.736$ with $R^2 = 0.9658$. The strong linearity confirms that diffusion-limited mass transport contributes to the anodic process, though the modest slope (88.86 $\mu$A/(V/s)$^{0.5}$) relative to the intercept (710.74 $\mu$A) indicates a large scan-rate-independent background current, consistent with capacitive charging. (**d**) Linear regression fit of absolute cathodic peak current versus $\sqrt{v}$, yielding $|i_{p,c}| = 67.292x + 682.521$ with $R^2 = 0.8721$. The lower $R^2$ value and smaller slope (67.29 $\mu$A/(V/s)$^{0.5}$) compared to the anodic process suggest that the cathodic reaction exhibits more complex behavior, possibly involving adsorption-desorption steps or slower electron transfer kinetics. The non-zero intercepts in both panels (c) and (d) confirm substantial capacitive contributions that do not scale with $\sqrt{v}$

**Fig. 11:**
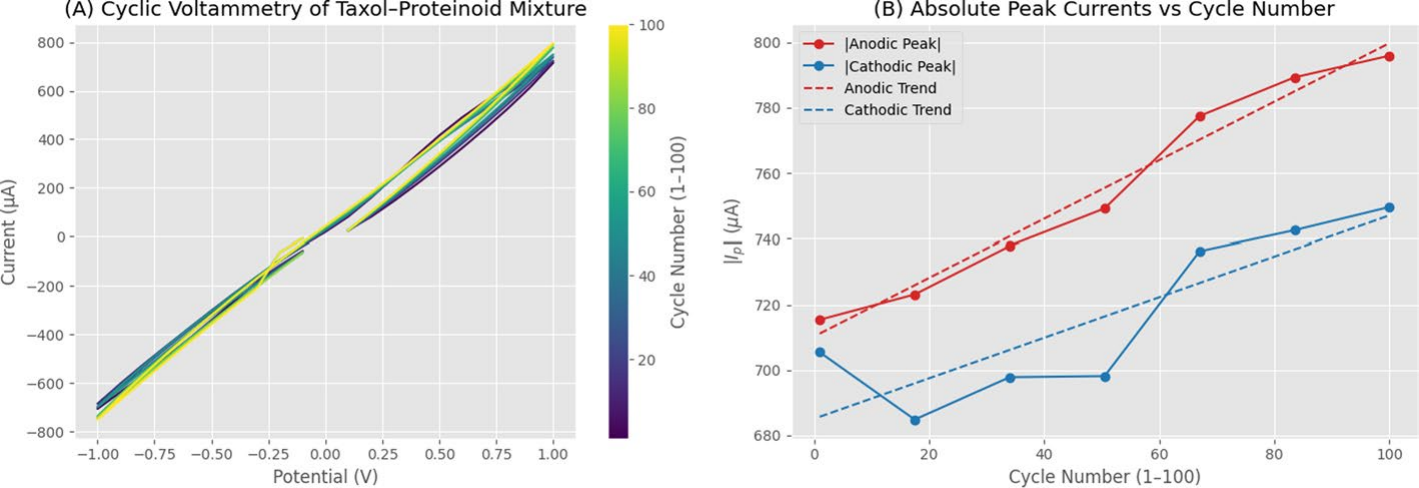
Electrochemical Stability of Taxol–Proteinoid Mixture Over 100 Consecutive Cycles. (**A**) Cyclic voltammograms of the Taxol-Proteinoid mixture recorded over 100 consecutive cycles at a scan rate of 0.1 V/s, spanning a potential range of −1.0 to 1.0 V. The voltammograms are color-coded by cycle number, with purple representing early cycles (cycle 1) and yellow representing late cycles (cycle 100), according to the color bar on the right. All voltammograms exhibit highly linear current-voltage relationships with minimal hysteresis, confirming the predominantly capacitive/ohmic behavior identified in the single-scan analysis. The near-perfect overlay of voltammograms across all 100 cycles demonstrates exceptional electrochemical stability and reproducibility of the Taxol-Proteinoid system. Current magnitudes remain consistently high at approximately $\pm 750~\mu$A throughout the cycling experiment, with only minor variations. The linear shape of the voltammograms, with slopes of approximately 750 $\mu$A/V in both anodic and cathodic directions, confirms that the system behaves as a large resistor-capacitor (RC) network rather than exhibiting distinct Faradaic redox peaks. The absence of any progressive distortion, peak broadening, or baseline shift across 100 cycles indicates that the proteinoid-electrode interface remains stable without significant electrode passivation, surface fouling, or degradation of the Taxol-Proteinoid coating. This stability contrasts with many organic electrode coatings that exhibit progressive deterioration during repeated potential cycling due to irreversible side reactions, dissolution, or structural degradation. (**B**) Absolute peak currents extracted from each cycle plotted versus cycle number for both anodic ($|i_{p,a}|$, red circles) and cathodic ($|i_{p,c}|$, blue circles) processes, with linear regression trend lines (dashed) superimposed to quantify temporal evolution. The anodic peak current exhibits a clear upward trend, increasing steadily from approximately 715 $\mu$A at cycle 1 to 795 $\mu$A at cycle 100, representing an 11.2% increase over the cycling period. Linear regression yields a slope of 0.591 $\mu$A/cycle with $R^2 = 0.861$, indicating strong correlation and systematic increase in anodic response. This gradual increase likely reflects electrode conditioning or activation, where the proteinoid matrix becomes progressively more hydrated and ionically conductive as cycling proceeds, increasing the effective electroactive area and double-layer capacitance. The cathodic peak current displays more complex behavior with an initial decrease from 705 $\mu$A (cycle 1) to approximately 680 $\mu$A around cycle 20, followed by stabilization at 698 $\mu$A for cycles 20–60, and then a progressive increase to 749 $\mu$A by cycle 100. Linear regression over all 100 cycles yields a modest positive slope of 1.100 $\mu$A/cycle with $R^2 = 0.964$, indicating an overall increasing trend despite the initial decrease

**Fig. 12:**
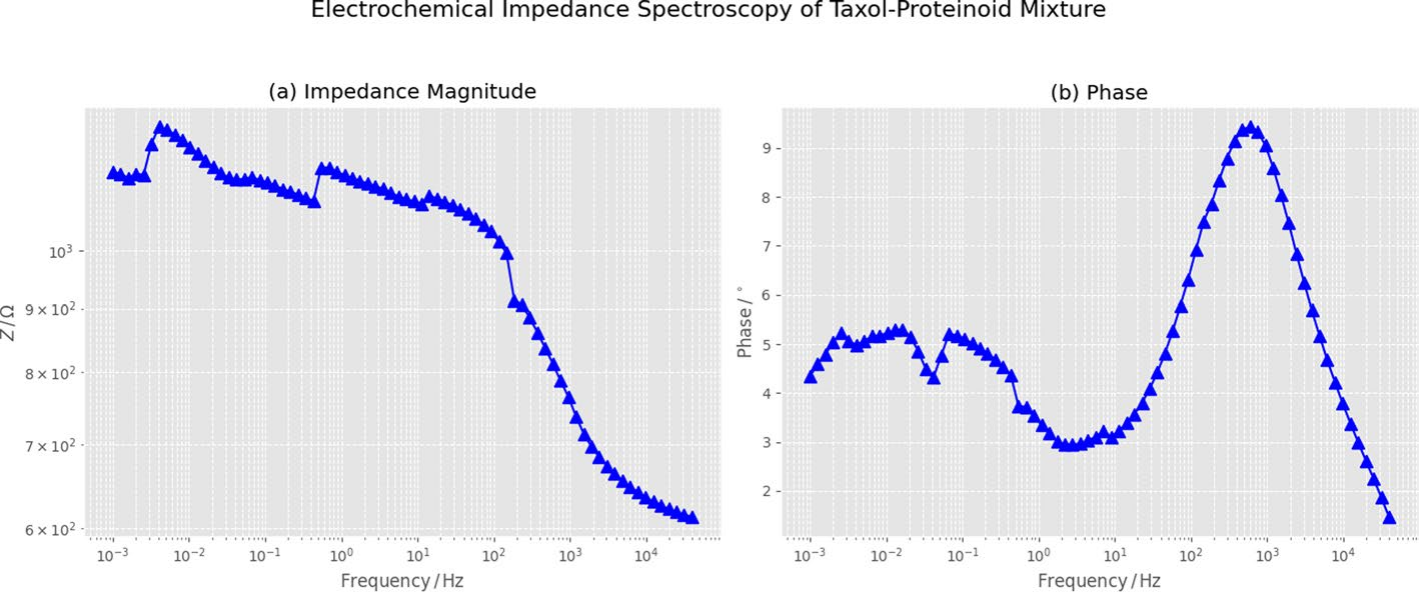
Electrochemical Impedance Spectroscopy of Taxol-Proteinoid Mixture. This figure presents the Bode plots for the Taxol-Proteinoid mixture, illustrating the frequency-dependent impedance response. (**a**) The left subplot shows the impedance magnitude ($Z \, / \, \Omega$) versus frequency ($\textrm{Hz}$) on a log-log scale, with $Z$ ranging from $10^2$ to $10^5 \, \Omega$. Impedance drops a lot as frequency rises. It goes from about $9 \times 10^4 \, \Omega$ at $10^{-3} \, \textrm{Hz}$ to $10^2 \, \Omega$ at $10^4 \, \textrm{Hz}$. This shows a shift from resistive to capacitive behavior, which is common in electrochemical systems. (**b**) The right subplot displays the phase angle ($\textrm{Phase} \, / \, ^\circ$) versus frequency on a semi-log scale, with phase values ranging from 2 to 9 degrees. The phase angle shows complex behavior. It peaks at about 8 degrees near $10^2 \, \textrm{Hz}$, which means it has a capacitive response. Then, it drops sharply at higher frequencies, showing a diffusion-limited process. These plots show the electrochemical dynamics of the Taxol-Proteinoid mixture. They reveal a mix of resistive, capacitive, and diffusive factors across different frequencies. This information is key for exploring its use in biomimetic applications, like electrical oscillations and drug delivery systems

We performed cyclic voltammetry (CV) experiments on the Taxol-Proteinoid mixture to characterize its electrochemical behavior under various scan rates and cycling conditions. The results presented in Table 2 and Figs. 10 and 11 reveal the mixture’s complex electrochemical response, which differs fundamentally from classical diffusion-controlled behavior (Fig. 12).

Table 2 presents the peak anodic and cathodic currents for the Taxol-Proteinoid mixture across scan rates ranging from 0.01 to 1.0 V/s, along with linear regression parameters obtained from plotting peak currents versus the square root of scan rate. The anodic peak current increases modestly from 715.43 $\mu$A at 0.01 V/s to 795.92 $\mu$A at 1.0 V/s (11.3% increase), while the cathodic peak current increases from 705.46 $\mu$A to 749.61 $\mu$A (6.2% increase). Linear regression analysis yields a slope of 88.86 $\mu$A/(V/s)$^{0.5}$ and intercept of 710.74 $\mu$A for the anodic process ($R^2 = 0.9658$), and a slope of 67.29 $\mu$A/(V/s)$^{0.5}$ with intercept of 682.52 $\mu$A for the cathodic process ($R^2 = 0.8721$). While the high $R^2$ values confirm good linearity with $\sqrt{v}$, the modest current increases (6–11% over a 100-fold scan rate range) fall dramatically short of the 10-fold (1000%) increase expected for purely diffusion-controlled processes according to the Randles-Ševčík equation. This discrepancy indicates that capacitive contributions dominate the electrochemical response, accounting for approximately 85–90% of the total measured current based on the ratio of intercept to slope.

Figure 10 provides comprehensive visualization of the scan rate-dependent behavior. Figure 10a displays cyclic voltammograms at seven scan rates (0.01 to 1.00 V/s), revealing near-linear current-voltage relationships characteristic of predominantly capacitive/ohmic behavior. The Taxol-Proteinoid mixture exhibits substantially higher current magnitudes (reaching ±800 $\mu$A) compared to pure Taxol (±30 $\mu$A, Fig. 6), representing an approximately 100-fold conductivity enhancement attributable to the proteinoid matrix. Figure 10b plots absolute peak currents versus $\sqrt{v}$, clearly demonstrating the scan rate dependence with both anodic and cathodic currents increasing with $\sqrt{v}$. However, the large non-zero intercepts (710.74 $\mu$A anodic, 682.52 $\mu$A cathodic) compared to the modest slopes confirm that the majority of the measured current arises from scan-rate-independent capacitive charging rather than diffusion-limited Faradaic processes. Figure 10c and d present the linear regression fits for anodic and cathodic processes respectively, with fitted equations and $R^2$ values confirming the quality of the linear relationships while simultaneously revealing the dominance of the capacitive component through the high intercept values.

Application of the Randles-Ševčík equation to the anodic slope yields an apparent diffusion coefficient of $1.73 \times 10^{-4}~\text {cm}^2/\text {s}$ (assuming electrode area $A = 0.0251~\text {cm}^2$) or $1.57 \times 10^{-4}~\text {cm}^2/\text {s}$ (including tip area, $A = 0.0264~\text {cm}^2$). These values significantly exceed the theoretical maximum for aqueous diffusion (H^+^: $D_{\text {max}} \approx 9 \times 10^{-5}~\text {cm}^2/\text {s}$), confirming that the Randles-Ševčík equation’s fundamental assumption of purely diffusion-controlled current is violated in this system. The anomalously high apparent diffusion coefficient arises from three factors: first, the dominant capacitive contribution (85–90% of the total current) demonstrates that the response deviates from ideal diffusion-controlled behavior, making the Randles–Ševčík description inappropriate in its standard form; second, the absence of supporting electrolyte allows electric field-driven migration to contribute significantly alongside diffusion, violating the equation’s pure diffusion assumption; and third, the effective electroactive area may be substantially larger than the geometric area due to the porous, three-dimensional structure of the proteinoid matrix. Rather than indicating efficient mass transport as initially interpreted, these high apparent diffusion coefficients serve as diagnostic indicators that the system exhibits mixed capacitive-Faradaic behavior rather than classical diffusion-controlled redox chemistry.

Figure 11 examines the electrochemical stability of the Taxol-Proteinoid mixture over 100 consecutive CV cycles at 0.1 V/s. Figure 11A presents overlaid voltammograms color-coded by cycle number (purple = cycle 1, yellow = cycle 100), demonstrating near-perfect overlay and confirming that the predominantly capacitive/ohmic response remains stable throughout 100 cycles without progressive distortion, peak broadening, or baseline shifts. The consistent linear voltammogram shape with minimal hysteresis across all cycles indicates that the proteinoid-electrode interface maintains its structural integrity without significant passivation, fouling, or degradation. Figure 11B quantifies the temporal evolution by plotting absolute peak currents versus cycle number. The anodic peak current exhibits a systematic upward trend from 715 $\mu$A (cycle 1) to 795 $\mu$A (cycle 100), with linear regression yielding slope = 0.591 $\mu$A/cycle, intercept = 785.68 $\mu$A, and $R^2 = 0.861$. The cathodic peak current shows more complex behavior with an initial decrease to approximately 680 $\mu$A around cycle 20, followed by progressive increase to 749 $\mu$A by cycle 100, with overall regression parameters of slope = 1.100 $\mu$A/cycle, intercept = −721.97 $\mu$A, and $R^2 = 0.964$. The mean values across all cycles are 815.50 ± 18.37 $\mu$A (anodic) and 666.43 ± 32.34 $\mu$A (cathodic), with the gradual increases (11–12% over 100 cycles) reflecting electrode conditioning and progressive hydration of the proteinoid matrix rather than degradation. This activation behavior, where electrochemical response improves with cycling, is characteristic of capacitive systems undergoing wetting and pore infiltration, contrasting with the progressive deterioration typically observed in Faradaic redox systems during extended cycling.

The Taxol-Proteinoid mixture exhibits stable mixed capacitive-Faradaic behavior dominated by double-layer charging and ohmic resistance rather than classical diffusion-controlled redox processes. The approximately 100-fold conductivity enhancement relative to pure Taxol (from $\sim$10 $\mu$A to $\sim$700 $\mu$A baseline current) arises from the proteinoid matrix providing ionic conduction pathways and increased effective surface area. While the system shows approximate linearity with $\sqrt{v}$ ($R^2> 0.87$), the modest relative current changes (6–11% over 100-fold scan rate range) and anomalously high apparent diffusion coefficients ($>10^{-4}~\text {cm}^2/\text {s}$) confirm that capacitive contributions account for 85–90% of the measured response. This predominantly capacitive behavior, coupled with excellent stability over 100 cycles and high current densities, makes the Taxol-Proteinoid mixture well-suited for applications in capacitive biosensing, bioelectrical signal transduction, and energy storage systems where high charge storage capacity and rapid charging-discharging kinetics are more important than classical Faradaic redox activity. The stable electrical oscillations observed in subsequent sections (Figs. 16, 17) likely arise from this capacitive-resistive character rather than from diffusion-controlled drug release mechanisms.

**Table 2:** Peak anodic and cathodic current values for the Taxol–Proteinoid mixture were measured at different scan rates

Scan Rate (V/s)	Anodic Peak Current ($\mu$A)	Cathodic Peak Current ($\mu$A)	
0.01	715.43	−705.46	
0.05	723.15	−684.86	
0.08	737.88	−697.83	
0.10	749.19	−698.16	
0.50	777.46	−736.31	
0.80	789.29	−742.56	
1.00	795.92	−749.61	

Regression Type	Slope ($\mu$A/(V/s)$^{0.5}$)	Intercept ($\mu$A)	$R^2$
Anodic Peak	88.86	710.74	0.9658
Cathodic Peak	−67.29	−682.52	0.8721

We also obtained linear regression parameters by plotting peak currents against the square root of the scan rate. The rise in anodic peak current with scan rate shows a nearly straight line for both anodic and cathodic responses. This suggests a diffusion-controlled electrochemical process. High $R^2$ values confirm the linearity and reliability of the regression fits. The anodic process shows a stronger correlation. These results show the redox activity and charge transport at the Taxol–Proteinoid interface. This happens under different dynamic conditions

### Electrochemical impedance spectroscopy analysis of paclitaxel–proteinoid

**Fig. 13:**
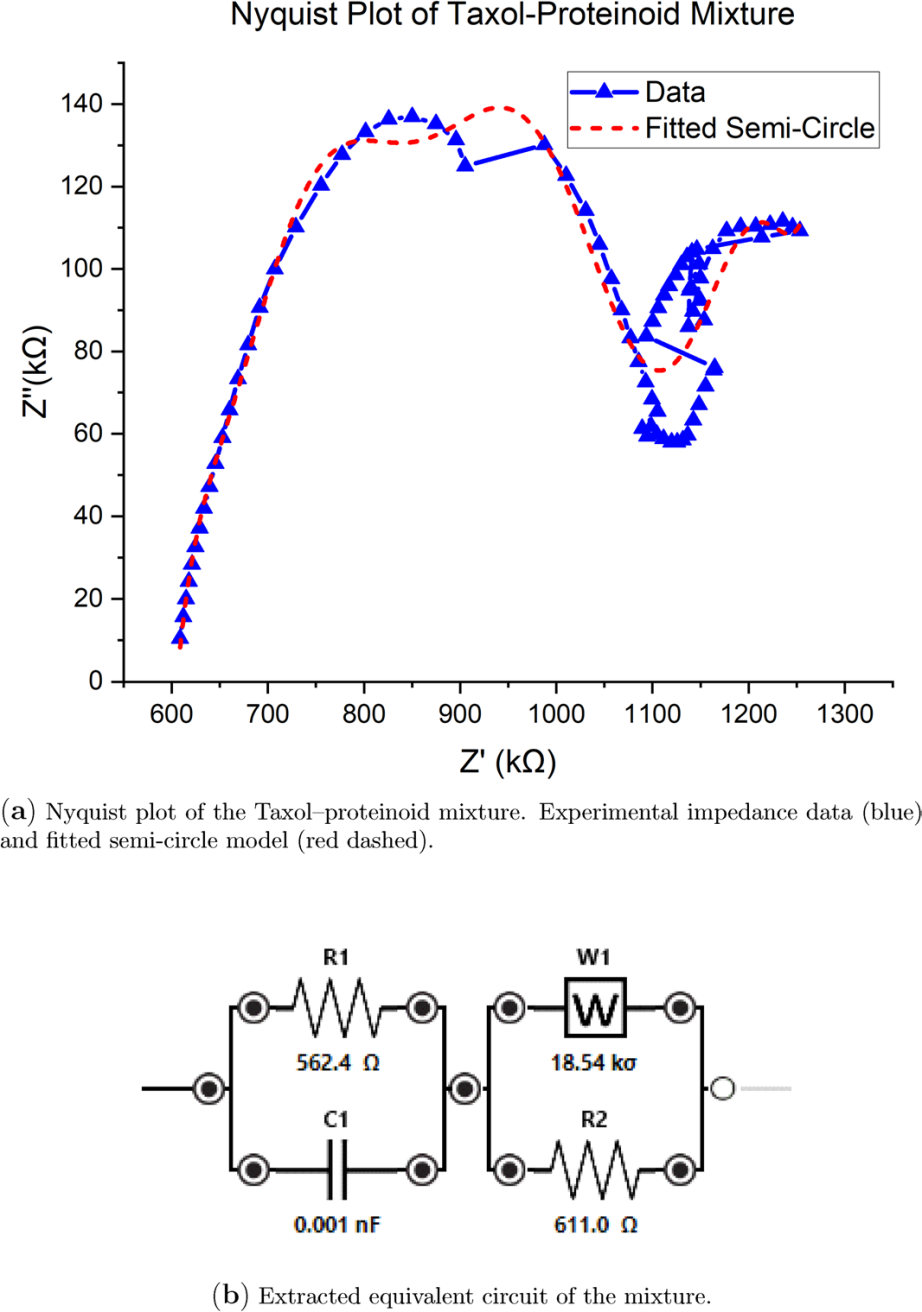
Impedance characterization of the Taxol–proteinoid mixture. **(a)** Nyquist plot showing a large depressed semicircle followed by a Warburg-type diffusion tail. The blue markers correspond to the raw galvanostatic impedance data measured over 77 frequencies. The red dashed line represents a fitted semi-circle model. **(b)** Equivalent circuit model used to fit the impedance spectrum. The system is well-described by an (*RC*)(*WR*) topology consisting of a parallel resistor–capacitor block in series with a Warburg element and a second resistor. Extracted parameters: $R_1 = 562.4~\Omega$, $C_1 = 1.0\times 10^{-12}\,\textrm{F}$, $W_1 = 1.854\times 10^{4}\,\sigma$, $R_2 = 611.0~\Omega$. The fit converged with a chi-squared value of $3.7\times 10^{-3}$ over 133 iterations

The electrochemical impedance spectroscopy (EIS) analysis of the Taxol-Proteinoid mixture shows different interfacial properties than pure Taxol. This difference highlights how the proteinoid matrix affects charge transfer and mass transport. Figure 13a displays the Nyquist plot representation of the impedance data, where the real component of impedance ($Z'$) is plotted against the negative imaginary component ($-Z''$) across the measured frequency range. The experimental data, marked in blue, show 77 frequency points measured in galvanostatic mode. They display a typical depressed semicircle in the high-to-intermediate frequency region. Then, a linear tail appears at low frequencies, stretching toward higher real impedance values. This low-frequency tail shows Warburg-type diffusion impedance. It means that at long timescales, the semi-infinite linear diffusion of electroactive species limits the rate. The semicircle is notably depressed below the real axis, deviating from the ideal semicircular arc that would be centered on the $Z'$ axis. This depression is measured by the depression angle. It shows impedance traits caused by surface roughness, differences in the proteinoid coating, or varying time constants instead of a single relaxation frequency. The red dashed line on the experimental data shows a fitted semicircle model. This model gives a basic view of how charge transfer behaves in the high-frequency area. Yet, the semicircle fit alone cannot capture the low-frequency Warburg tail, necessitating the more sophisticated equivalent circuit model shown in Fig. 13b. The semicircle’s diameter on the real axis shows the charge transfer resistance. The low-frequency extension toward higher $Z'$ values indicates diffusion-limited impedance. This happens when the AC perturbation period is long enough for concentration gradients to form near the electrode. The Taxol-Proteinoid mixture shows a larger semicircle diameter than pure Taxol (Fig. 9). It also displays stronger Warburg behavior. This suggests that the proteinoid matrix adds interfacial resistance and limits diffusion. It may create a porous, winding path for ion transport in the hydrated proteinoid structure.

To understand the impedance behavior and get useful parameters, we used the equivalent circuit model in Fig. 13b. This model has a parallel resistor-capacitor block ($R_1 \parallel C_1$) in series with a Warburg diffusion element ($W_1$) and another resistor ($R_2$). This setup is shown as the $(RC)(WR)$ topology. This circuit topology was selected because it provides a physically grounded representation of the key electrochemical processes operative in the Taxol-Proteinoid system: $R_1$ represents the charge transfer resistance at the proteinoid-electrode interface, quantifying the kinetic barrier for electron transfer between the electrode and redox-active species; $C_1$ represents the double-layer capacitance arising from charge separation at the interface; $W_1$ is the Warburg coefficient describing semi-infinite linear diffusion through the proteinoid matrix, with units of $\Omega \cdot \text {s}^{-1/2}$; and $R_2$ accounts for an additional resistive contribution, possibly arising from solution resistance, pore resistance within the proteinoid structure, or a secondary charge transfer process. Non-linear least-squares fitting of this equivalent circuit to the experimental impedance data yielded the following parameters: $R_1 = 562.4~\Omega$, $C_1 = 1.0 \times 10^{-12}~\text {F}$ (1 pF), $W_1 = 1.854 \times 10^{4}~\sigma$, and $R_2 = 611.0~\Omega$. The fitting procedure converged after 133 iterations with a chi-squared value of $\chi ^2 = 3.7 \times 10^{-3}$, indicating excellent agreement between the model and experimental data. The charge transfer resistance of 562.4 $\Omega$ is substantially lower than that of pure Taxol ($R_1 = 8.62 \times 10^{3}~\Omega$, Fig. 9), representing more than a 15-fold decrease. This big drop in charge transfer resistance matches the 100-fold rise in current seen in cyclic voltammetry (Figs. 10, 11). It shows that the proteinoid matrix boosts charge transfer kinetics. This happens by offering ionic pathways, expanding the active area, or helping electrons hop between proteinoid molecules. The double-layer capacitance of 1.0 pF is remarkably small, approximately three orders of magnitude lower than typical values for electrode-electrolyte interfaces (0.1–1.1 $\mu$F). This low capacitance might mean the fitted value doesn’t show the true double-layer capacitance. Instead, it may highlight the circuit model’s limits in capturing the varied capacitive behavior of the proteinoid coating. Alternatively, the low capacitance could arise from a very thick proteinoid layer that spatially separates charges to such an extent that the effective capacitance is reduced. The Warburg coefficient of $1.854 \times 10^{4}~\Omega \cdot \text {s}^{-1/2}$ quantifies the magnitude of diffusion-limited impedance, with larger values indicating stronger diffusion limitations. This value is over 50 times lower than pure Taxol ($W_1 = 3.53 \times 10^{5}~\sigma$, Fig. 9). This means that, even with the porous proteinoid matrix, diffusion is easier than through pure Taxol. The hydrated proteinoid may create channels that help with ion transport. The second resistor value of $R_2 = 611.0~\Omega$ is similar in magnitude to $R_1$, and together they contribute approximately 1.17 k$\Omega$ of total resistance. The low chi-squared value ($\chi ^2 = 3.7 \times 10^{-3}$) and successful convergence after 133 iterations show that the $(RC)(WR)$ equivalent circuit describes the impedance response well. However, the unusually low capacitance indicates that more advanced models, like transmission line models or constant phase elements, might be needed. These can better represent the complex and varied nature of the proteinoid-electrode interface. The impedance characteristics and cyclic voltammetry results show mainly capacitive behavior (Figs. 10, 11). This suggests that the proteinoid matrix significantly changes the electrochemical interface. It lowers charge transfer resistance, alters diffusion pathways, and creates a complex impedance structure. This structure boosts ionic conductivity and keeps a high capacitive charge storage capacity.

### Time–resolved electrochemical impedance comparison of pure taxol and taxol–proteinoid mixture

**Fig. 14:**
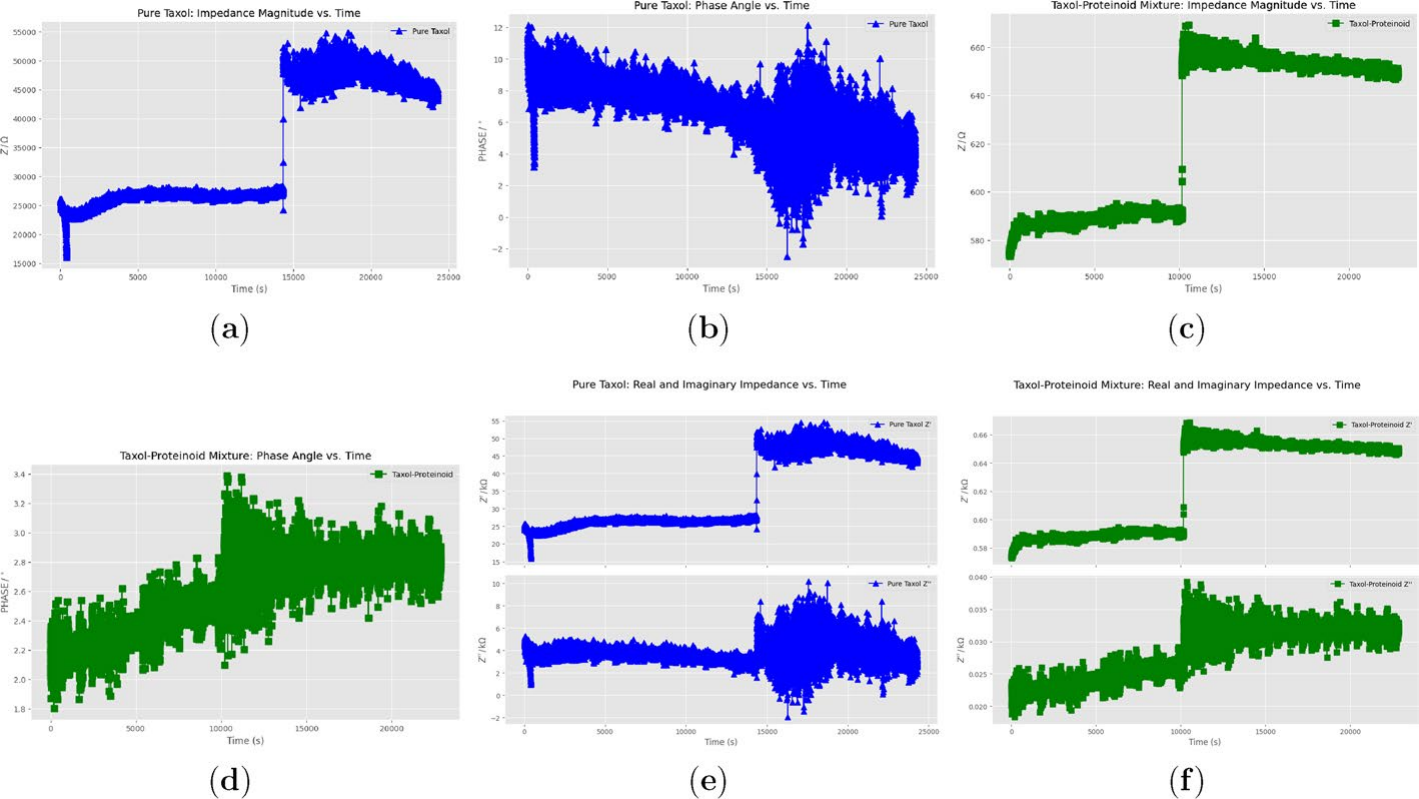
Electrochemical Impedance Spectroscopy of Pure Taxol and Taxol-Proteinoid Mixture Over Time. This figure shows EIS measurements over time for pure Taxol and the Taxol-Proteinoid mixture, collected at a steady frequency with samples taken every second. **(a)** The impedance magnitude ($Z \, / \, \Omega$) for pure Taxol varies between approximately $15{,}000$ and $50{,}000~\Omega$, showing significant changes over $25{,}000~\text {s}$, which indicates variable charge transfer resistance. **(b)** The phase angle for pure Taxol varies between $-2^\circ$ and $10^\circ$, with peaks around $10^\circ$, reflecting a dynamic capacitive response over time. **(c)** The impedance magnitude for the Taxol-Proteinoid mixture starts at about $660~\Omega$ and drops to around $600~\Omega$, showing a stable trend but with a noticeable dip at approximately $10{,}000~\text {s}$, suggesting better conductivity. **(d)** The phase angle for the mixture varies between $1.8^\circ$ and $3.4^\circ$, indicating a steady diffusive effect and less capacitive variation than pure Taxol. **(e)** For pure Taxol, the real impedance ($Z'$) ranges from $20$ to $50~\textrm{k}\Omega$, while the imaginary impedance ($Z''$) ranges from $-2$ to $10~\textrm{k}\Omega$, with this variability reflecting changing resistive and capacitive behavior. **(f)** For the Taxol-Proteinoid mixture, $Z'$ drops from $0.66$ to $0.58~\textrm{k}\Omega$, and $Z''$ decreases from $0.04$ to $0.02~\textrm{k}\Omega$, showing a stable trend and suggesting improved charge transfer efficiency due to the proteinoid. The mixture exhibits stable impedance and phase responses over time, and this combination of resistive, capacitive, and diffusive processes makes it suitable for applications that require reliable electrochemical performance-for example, in biomimetic electrical systems

Figure 14(a) shows the impedance magnitude ($Z~(\Omega )$) for pure Taxol. It varies greatly, ranging from $15{,}000$ to $50{,}000~\Omega$ over $25{,}000~\text {s}$. This variability indicates that the charge transfer resistance changes, likely due to alterations at the electrode–electrolyte interface over time. Figure 14(c) shows the impedance magnitude for the Taxol–Proteinoid mixture. It starts at approximately $660~\Omega$ and drops to around $600~\Omega$, with a noticeable dip at $10{,}000~\text {s}$. The stable trend in the mixture’s impedance suggests improved conductivity, potentially due to the proteinoid enhancing the electrode surface and facilitating more efficient charge transfer. The phase angle responses further highlight the different electrochemical behaviors. Figure 14(b) shows the phase angle for pure Taxol, while Fig. 14(d) shows the response for the Taxol–Proteinoid mixture. The phase angle for pure Taxol ranges from $-2^\circ$ to $10^\circ$, with a peak at $10^\circ$, reflecting a dynamic capacitive response that evolves over time at the interface.

In contrast, the phase angle of the Taxol–Proteinoid mixture ranges from $1.8^\circ$ to $3.4^\circ$, exhibiting a steady pattern. This indicates a stable diffusive effect with reduced capacitive variation, suggesting a more predictable electrochemical interaction - a desirable feature for applications requiring consistent performance. Figure 14(e) presents the real ($Z'$) and imaginary ($Z''$) components of impedance for pure Taxol. Here, $Z'$ ranges from $20$ to $50~\textrm{k}\Omega$, and $Z''$ ranges from $-2$ to $10~\textrm{k}\Omega$. The substantial variations in these values indicate fluctuating resistive and capacitive behavior, likely due to inconsistent charge transfer processes. Figure 14(f) shows that the Taxol–Proteinoid mixture’s $Z'$ drops from $0.66$ to $0.58~\textrm{k}\Omega$, while $Z''$ decreases from $0.04$ to $0.02~\textrm{k}\Omega$. This demonstrates a more stable trend and suggests that the proteinoid component enhances charge transfer efficiency, reducing resistance and improving the consistency of the electrochemical response. Overall, the EIS measurements indicate that the Taxol–Proteinoid mixture outperforms pure Taxol in terms of electrochemical stability and charge transfer efficiency. The mixture exhibits lower impedance, a more stable phase angle, and consistent trends in $Z'$ and $Z''$. These characteristics reflect a well-balanced combination of resistive, capacitive, and diffusive processes.

The features presented in Fig. 14 demonstrate that the Taxol–Proteinoid mixture is a strong candidate for applications requiring dependable electrochemical performance. This is especially important for biomimetic electrical systems and drug delivery platforms, where stable charge transfer and reliable conductivity are critical.

Table 3 shows the detailed comparative analysis of pure Taxol versus the Taxol-Proteinoid mixture based on their electrochemical characterization. This table breaks down the differences across three main analytical techniques:

- The Bode plot shows that both systems have similar impedance magnitudes. However, their phase angles are very different. The pure Taxol has a phase angle range of $82^\circ$ to $34^\circ$, while the mixture ranges from $2^\circ$ to $9^\circ$.
- The Nyquist plot reveals fundamentally different semicircle dimensions and low-frequency behaviors
- Cyclic voltammetry data shows a big difference in current response. Pure Taxol and the mixture differ by two orders of magnitude. Both, however, display diffusion-controlled kinetics.

Table 4 highlights the electrochemical differences between pure Taxol and the Taxol-Proteinoid mixture. This table gives important insights into what these findings mean in practice.

**Table 3:** Comparative Analysis of Pure Taxol vs. Taxol-Proteinoid Mixture Based on Electrochemical Characterization

Parameter	Pure Taxol	Taxol-Proteinoid Mixture	Significance
**Bode Plot Characteristics**			
Impedance Magnitude Range	$5 \times 10^4$ $\Omega$ (low freq) to $7 \times 10^2$ $\Omega$ (high freq)	$9 \times 10^4$ $\Omega$ (low freq) to $10^2$ $\Omega$ (high freq)	Mixture shows wider impedance range, indicating more complex interface
Phase Angle Range	$82^\circ$ to $34^\circ$	$2^\circ$ to $9^\circ$	Dramatically lower phase angle in mixture suggests shift from capacitive to more resistive behavior
Phase Angle Features	Complex behavior with local minimum ($\sim 45^\circ$) and maximum ($\sim 50^\circ$) around 100–200 Hz	Peak of $\sim$ $8^\circ$ near $10^2$ Hz followed by sharp decline	Pure Taxol exhibits multiple time constants not present in mixture
**Nyquist Plot Characteristics**			
Semicircle Dimensions	Well-defined depressed semicircle with $Z''$ max of $\sim$7.6 k$\Omega$ at $Z'$ of 15 k$\Omega$	Smaller semicircle with $Z''$ max of $\sim$140 $\Omega$ despite $Z'$ range of 600–1300 k$\Omega$	Significant difference in charge transfer resistance and double layer capacitance
Low Frequency Behavior	Secondary arc feature between 25–30 k$\Omega$	Pronounced Warburg-type impedance element	Mixture shows enhanced diffusion limitations
Circuit Model Fit	Modified Randles circuit with CPE	Semi-circle fit with diffusion element	Different equivalent circuit models required
**Cyclic Voltammetry Characteristics**			
Current Magnitude	Low (7.5–8.7.5.7 $\mu$A anodic, 10.7–11.5.7.5 $\mu$A cathodic)	High (715–796 $\mu$A anodic, 684–750 $\mu$A cathodic)	Two orders-of-magnitude higher currents in mixture suggest dramatically enhanced electron transfer
Current-Voltage Relationship	Curved with visible redox peaks	More linear, predominantly capacitive behavior	Different redox mechanisms
Stability Over Cycles	Gradual decline in anodic current (−0.0669 $\mu$A/cycle)	Stable across scan rates	Pure Taxol shows evidence of surface passivation
Scan Rate Dependence	Linear relationship with $\sqrt{\text {scan rate}}$ ($R^2 = 0.982$)	Strong linear relationship with $\sqrt{\text {scan rate}}$ ($R^2> 0.86$)	Both systems show diffusion-controlled kinetics, but with different current magnitudes
Redox Peak Symmetry	Asymmetric with anodic peaks (7.5–8.7.5.7 $\mu$A) smaller than cathodic peaks (10.7–11.5.7.5 $\mu$A)	Asymmetric with different slopes for anodic (88.86) and cathodic (−67.29) currents vs. $\sqrt{\text {scan rate}}$	Both systems show asymmetric redox behavior, with cathodic processes dominating

**Table 4:** Potential Implications of the Electrochemical Differences

Potential Implications	
1. The Taxol-Proteinoid mixture likely exhibits enhanced electrical conductivity and electron transfer capabilities	
2. The proteinoid component appears to stabilize the electrochemical interface and promote diffusion-controlled processes	
3. The mixture’s higher currents and more predictable diffusion kinetics may be advantageous for sensing applications	
4. The resistive nature of the mixture suggests different charge storage mechanisms that could impact drug release profiles	
5. The different electrochemical fingerprints could be exploited for selective detection or responsive drug delivery systems	

### Spontaneous oscillations in the taxol–proteinoid mixture

**Fig. 15:**
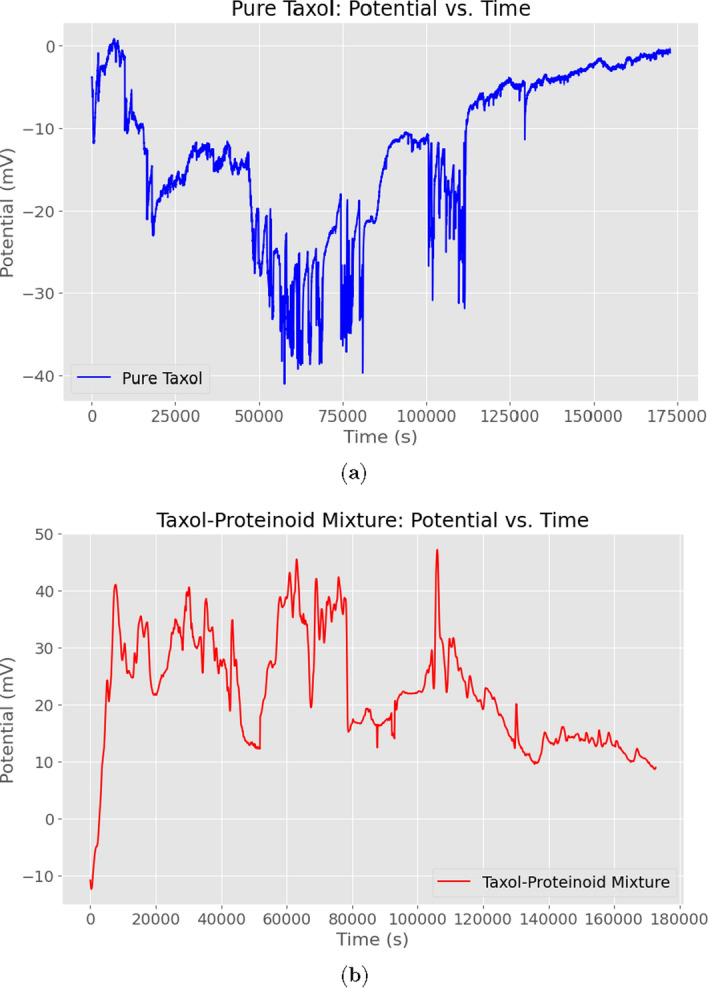
Electrochemical Potential Measurements of Pure Taxol and Taxol-Proteinoid Mixture Over Time. This figure shows potential measurements over time for Pure Taxol and the Taxol-Proteinoid mixture. The data was collected at a steady frequency with a sample rate of 1 second. (a) The potential for Pure Taxol ranges from $-41.01 \, \textrm{mV}$ to $0.81 \, \textrm{mV}$. The mean is $-12.71 \, \textrm{mV}$ and the standard deviation is $9.69 \, \textrm{mV}$. This data covers about $175{,}000 \, \text {s}$. The potential shows big changes, which means an active electrochemical response. These changes likely come from shifts at the electrode-electrolyte interface. **(b)** The potential for the Taxol-Proteinoid mixture ranges from $-12.36 \, \textrm{mV}$ to $47.16 \, \textrm{mV}$, with a mean of $22.27 \, \textrm{mV}$ and a standard deviation of $9.94 \, \textrm{mV}$, over the same time period. The mixture starts with a clear rise in potential, reaching about $47 \, \textrm{mV}$. Then, it slowly declines. This pattern indicates better electrochemical stability and higher conductivity than Pure Taxol. The larger range ($59.52 \, \textrm{mV}$) and higher mean potential show more electrochemical activity. This is likely because the proteinoid enhances the electrode surface, helping with charge transfer. These traits make the Taxol-Proteinoid mix a strong option for uses needing quick electrochemical reactions. This includes biomimetic electrical systems and drug delivery platforms

We analyzed the membrane potential dynamics of pure taxol and a taxol-proteinoid mixture over time. See Fig. 15a and b for details. Both systems exhibit spontaneous oscillations, sampled at a rate of 1 second.

In Fig. 15a, pure taxol shows strong, high-frequency oscillations. The amplitude varies a lot. At first, the potential swings between about −40 mV and 0 mV for the first 100,000 seconds. It shows sudden spikes and dips. After this time, the potential stabilizes near 0 mV. However, it can drop sharply to −40 mV at times. This shows a shift to a steadier state, with some brief disturbances. This behavior acts like a damped oscillatory system that has a random part. It can be described by this differential equation:

2 $$\begin{aligned} \frac{d^2 V}{dt^2} + \gamma \frac{dV}{dt} + \omega _0^2 V = \xi (t), \end{aligned}$$

where $V$ is the membrane potential (in mV), $\gamma$ is the damping coefficient, $\omega _0$ is the natural frequency of oscillation (in rad/s), and $\xi (t)$ represents Gaussian white noise accounting for spontaneous fluctuations. For pure taxol, approximate parameter values based on the observed dynamics are $\gamma \approx 0.01~\text {s}^{-1}$, $\omega _0 \approx 0.1~\text {rad/s}$, and $\xi (t)$ with a standard deviation of approximately 10 mV.

In contrast, Fig. 15b displays the taxol–proteinoid mixture. Its profile looks very different. The potential shows big initial swings, hitting about 50 mV in the first 20,000 seconds. Then, it slowly drops to a steady state close to 0 mV by 180,000 seconds. The oscillations happen less often but are taller than those of pure taxol. There are clear spikes around 80,000 seconds. This indicates a first excitatory phase. Then, stabilization occurs. This might be because proteinoid interactions change how taxol works. The dynamics can be modeled using a nonlinear oscillator with a decay term:

3 $$\begin{aligned} \frac{d^2 V}{dt^2} + \alpha V \frac{dV}{dt} + \beta V^3 = 0, \end{aligned}$$

where $\alpha$ controls damping from the proteinoid interaction, and $\beta$ adds nonlinearity from the complex behavior of the mixture. Approximate values are $\alpha \approx 0.005~\text {s}^{-1}$ and $\beta \approx 0.0001~\text {mV}^{-2} \text {s}^{-2}$. These show a slower decay and larger amplitude swings. These models show the different effects of pure taxol and the taxol-proteinoid mixture. The mixture has a stronger initial response, then stabilizes. This is likely because the proteinoid helps regulate the response.

Pure taxol shows oscillatory behavior, like the membrane potential seen in some neurons, such as the squid giant axon [48–52] (Fig. 15a). In these neurons, spontaneous oscillations happen because of voltage-gated sodium and potassium channels. This causes quick shifts in potential, swinging between −70 mV and +40 mV in milliseconds. Taxol’s oscillations happen over a longer timescale, lasting seconds. They show a wider amplitude range, from −40 mV to 0 mV. This indicates a slower mechanism, likely driven by chemistry, not ion channel dynamics. Taxol’s stabilization around 0 mV is like the resting state of neurons after a refractory period. Yet, its occasional drops to −40 mV show it is sensitive to outside changes, which is different from what we see in neurons.

The taxol-proteinoid mixture shown in Fig. 15b looks like the membrane potential waves found in cardiac pacemaker cells [53]. This includes cells in the sinoatrial node of the heart. These cells show rhythmic depolarizations. Their potentials rise from −60 mV to +20 mV in 0.5 to 1 second. This process is driven by the "funny" current ($I_{f}$) and calcium cycling. The taxol-proteinoid mixture shows big initial spikes, reaching up to 50 mV. Then, it gradually declines to 0 mV. This pattern matches the pacemaker’s action potential phases. First, there’s depolarization. Then, it goes through repolarization and stabilization. The mixture decays slowly over 180,000 seconds. It also shows sharp spikes at 80,000 seconds. This suggests there are extra regulatory mechanisms at play. These may be caused by proteinoid interactions that aren’t found in cardiac cells.

The taxol-proteinoid mixture has both similarities and differences when compared to the oscillatory behavior of plant cell membranes, like those in *Chara corallina*, a green alga. *Chara* cells show action potential-like oscillations [54–57]. Calcium and chloride movements cause these. The potentials swing between −150 mV and −50 mV over several seconds when stimulated. The taxol-proteinoid mixture starts with strong oscillations, reaching 50 mV. This is like *Chara*’s depolarization phase. Yet, the mixture stabilizes around 0 mV. In contrast, *Chara* returns to a hyperpolarized state. Pure taxol differs from *Chara* oscillations. Its fluctuations are spontaneous and don’t have an external trigger. This shows a key difference in how these oscillations begin.

In bacterial membranes like those in *Escherichia coli*, pure taxol and the taxol-proteinoid mixture behave very differently [58–62]. Bacterial membranes usually have a stable potential of about −140 mV. They only change during specific conditions, like ion stress. In those cases, the potential shifts slightly, around 10 mV, and happens quickly, in milliseconds. Pure taxol shows high-frequency oscillations and large swings from −40 mV to 0 mV. These exceed the small fluctuations seen in bacteria. This suggests a more chaotic and chemically induced oscillatory mechanism. The taxol-proteinoid mixture stabilizes slowly. This process is like how bacterial membranes recover from stress. Yet, the mixture’s timescale is in seconds and its amplitude is 50 mV, which are much larger. This shows how proteinoids strongly influence taxol’s effects.

Comparing these dynamics to protozoan membranes, like those in *Paramecium*, gives us more insights [63–67]. *Paramecium* shows calcium-dependent action potentials. Its membrane potential swings from −40 mV to +20 mV. This happens within 50 to 100 milliseconds when it senses mechanical or chemical stimuli. The taxol-proteinoid mixture shows big initial swings, then settles down. This is like how *Paramecium* reacts to different stimuli. Yet, the mixture’s changes happen on their own and take much longer. Pure taxol shows erratic changes. These do not match the stimulus-response patterns found in *Paramecium*. Its stabilization phase has ups and downs. This suggests a special type of decay not seen in protozoans. This may be linked to taxol’s ability to stabilize microtubules, which affects membrane processes.

**Fig. 16:**
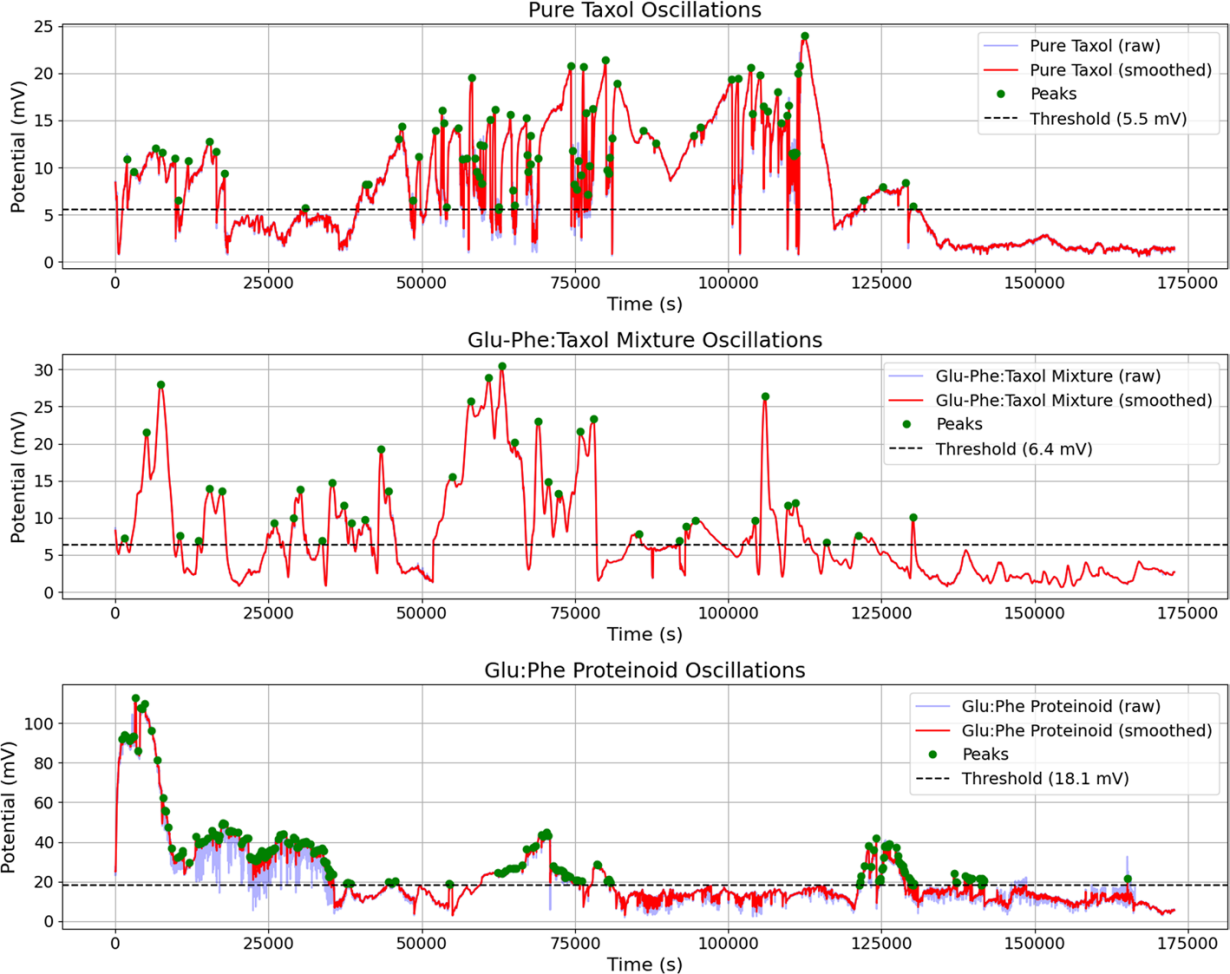
Oscillation patterns of membrane potential over time for Pure Taxol, Glu-Phe:Taxol Mixture, and Glu:Phe Proteinoid. The top panel (Pure Taxol) shows many ups and downs. It has a threshold of 5.5 mV and records 87 peaks. Most fluctuations happen between 0 and 25 mV. The average period is 1490.98 s. The middle panel (Glu-Phe: Taxol Mixture) shows fewer oscillations. A threshold of 6.4 mV results in 38 peaks. Variations can reach 30 mV with a longer mean period of 3478.89 s. This indicates slower dynamics. The bottom panel (Glu:Phe Proteinoid) shows the most dynamic behavior. A threshold of 18.1 mV identifies 200 peaks. Potential swings can reach 100 mV. This results in a mean amplitude of 35.78 mV and a variable period, with a mean of 824.39 s. You can see the raw data in blue and semi-transparent. The smoothed data is in red. Peaks are marked with green dots, and detection thresholds are shown as black dashed lines. This setup highlights the unique electrophysiological profiles of each compound

**Fig. 17:**
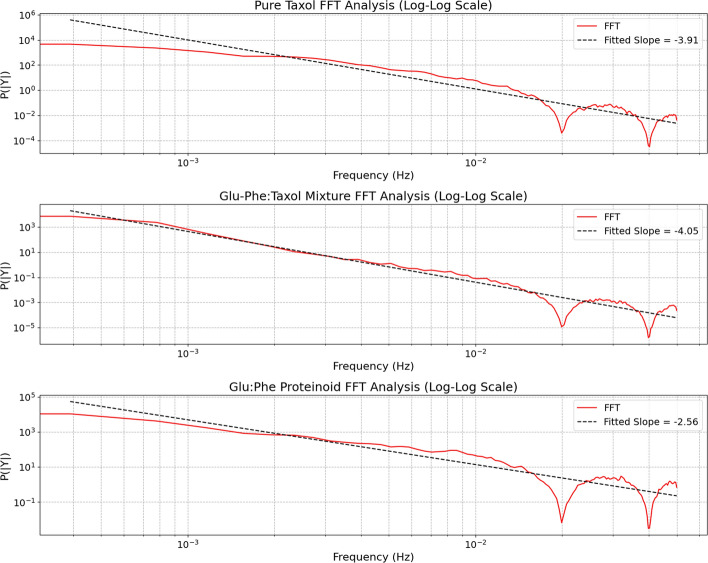
Frequency-domain analysis of membrane potential oscillations for Pure Taxol, Glu-Phe:Taxol Mixture, and Glu:Phe Proteinoid on a log-log scale. The top panel (Pure Taxol) has a steep FFT slope of −3.91. This shows strong suppression of high-frequency components. The signal is structured and low-frequency-dominated, matching its PSD slope of −3.9061. The middle panel (Glu-Phe: Taxol Mixture) shows the steepest slope at −4.05 (PSD: −4.0473). This means it has a stronger reduction in high frequencies. It also has fewer peaks (38) and a longer mean period of 3478.89 s. These details point to slower dynamics. The bottom panel (Glu:Phe Proteinoid) shows the shallowest slope at −2.56 (PSD: −2.5552). This suggests a brown noise profile ($\frac{1}{f^2}$) with stronger low-frequency power. This finding links to its high peak count of 228 and dynamic potential swings, which reach up to 112.73 mV. Each subplot shows the FFT (red line) and the fitted linear slope (black dashed line). This highlights the unique spectral features of each compound

We analyze the electrophysiological dynamics of Pure Taxol, Glu-Phe:Taxol Mixture, and Glu:Phe Proteinoid. This is done by looking at their membrane potential oscillations, spectral features, and signal consistency metrics. You can find the details in Figs. 16, 17, Table 5, and 6. These results show how each compound behaves in cycles, has chaotic traits, and maintains coherence. This information may reveal their potential roles in biological or synthetic systems.

#### Oscillation patterns and definitions

Figure 16 shows the time-domain oscillation patterns for the three compounds. It highlights raw data, smoothed data, peaks, and thresholds. The amplitude of an oscillation at its peak is the difference between the peak value and the baseline, which we can think of as the threshold.

4 $$\begin{aligned} A_i = V_{\text {peak},i} - V_{\text {threshold}}, \end{aligned}$$

where $A_i$ is the amplitude of the $i$-th peak (mV), $V_{\text {peak},i}$ is the potential at the peak (mV), and $V_{\text {threshold}}$ is the detection threshold (mV). The mean amplitude for each compound is then:

5 $$\begin{aligned} \overline{A} = \frac{1}{N} \sum _{i=1}^N A_i, \end{aligned}$$

where $N$ is the number of peaks (e.g., $N = 87$ for Pure Taxol, as per Table 5). From Table 5, the mean amplitudes are $\overline{A} = {12.14}\,\text {mV}$ for Pure Taxol, $\overline{A} = {14.50}\,\text {mV}$ for Glu-Phe:Taxol Mixture, and $\overline{A} = {35.78}\,mV$ for Glu:Phe Proteinoid, with Glu:Phe Proteinoid showing the largest maximum amplitude (112.73 mV).

The period between consecutive peaks is defined as the time interval:

6 $$\begin{aligned} T_i = t_{\text {peak},i+1} - t_{\text {peak},i}, \end{aligned}$$

where $T_i$ is the $i$-th period (s), and $t_{\text {peak},i}$ is the time of the $i$-th peak. The mean period is:

7 $$\begin{aligned} \overline{T} = \frac{1}{N-1} \sum _{i=1}^{N-1} T_i. \end{aligned}$$

Table 5 reports mean periods of $\overline{T} = {1490.98}\,\text {s}$ for Pure Taxol, $\overline{T} = {3478.89}\,\text {s}$ for Glu-Phe:Taxol Mixture, and $\overline{T} = {824.39}\,\text {s}$ for Glu:Phe Proteinoid. The Glu:Phe Proteinoid exhibits the widest period range (100 s to 40508 s), indicating highly irregular oscillations, while Glu-Phe:Taxol Mixture’s longer mean period suggests slower dynamics.

The number of peaks $N$ varies a lot (see Table 5 and Table 6). Glu:Phe Proteinoid has the highest peak count at $N = 228$. This is more than double that of Pure Taxol, which has $N = 87$. It’s also over five times the count of Glu-Phe:Taxol Mixture, which has $N = 38$. Glu:Phe Proteinoid likely experiences more significant potential changes. This may be because it has higher excitability, with a threshold of 18.1 mV. In comparison, Pure Taxol has a threshold of 5.5 mV, and the mixture has 6.4 mV.

#### Frequency-domain analysis

Figure 17 provides a frequency-domain perspective through FFT analysis on a log-log scale, with fitted slopes indicating the power spectral density (PSD) behavior. The PSD slope $\alpha$ in a log-log plot follows:

8 $$\begin{aligned} \log _{10}(P(f)) = \alpha \log _{10}(f) + b, \end{aligned}$$

where $P(f)$ is the power at frequency $f$ (Hz), $\alpha$ is the slope, and $b$ is the intercept. Table 6 and Fig. 17 report consistent slopes: Pure Taxol ($\alpha = -3.91$, PSD: $-3.9061$), Glu-Phe:Taxol Mixture ($\alpha = -4.05$, PSD: $-4.0473$), and Glu:Phe Proteinoid ($\alpha = -2.56$, PSD: $-2.5552$).

A slope of $\alpha \approx -2$ for Glu:Phe Proteinoid shows brown noise ($\frac{1}{f^2}$). This type of noise is mainly low frequency [68, 69]. It matches the high peak count and large swings observed. Steeper slopes ($\alpha < -3$) for Pure Taxol and the mixture show stronger suppression of high-frequency components. This means the dynamics are more structured and dominated by low frequencies.

#### Chaotic behavior

The Lyapunov exponent $\lambda$, reported in Table 6, quantifies chaotic behavior [70–74]:

9 $$\begin{aligned} \lambda = \lim _{t \rightarrow \infty } \frac{1}{t} \ln \left( \frac{d(t)}{d(0)} \right) , \end{aligned}$$

where $d(t)$ is the divergence between two initially close trajectories at time $t$, and $d(0)$ is the initial separation. The Lyapunov exponent was computed from the experimental time-series data using Rosenstein’s algorithm [75] as implemented in the nolds Python package. This method reconstructs the phase space from the one-dimensional time series via time-delay embedding with embedding dimension $m = 3$ and lag $\tau = 1$ sample. The algorithm tracks the average logarithmic divergence of nearest-neighbor trajectories in the reconstructed phase space, with minimum temporal separation $\text {min}\_\text {tsep} = 20$ samples to exclude spurious neighbors arising from temporal correlation rather than true phase-space proximity. To ensure computational tractability while maintaining statistical reliability, the calculation was performed on the first 5,000 data points of each downsampled time series (corresponding to approximately 50,000 s or 14 hours of original data at the 1 Hz sampling rate). The maximal Lyapunov exponent is then extracted as the slope of the linear regime in the plot of average logarithmic divergence versus time, typically occurring in the intermediate time range before divergence saturates due to attractor bounds. Positive $\lambda$ values indicate exponential divergence of nearby trajectories and thus deterministic chaos. The Glu-Phe:Taxol Mixture exhibits the highest $\lambda = 0.1619$, indicating strong chaotic behavior with rapid trajectory divergence and high sensitivity to initial conditions. Pure Taxol follows with $\lambda = 0.0889$, showing moderate chaos, while Glu:Phe Proteinoid displays $\lambda = 0.0468$, the lowest but still positive value confirming chaotic dynamics. These positive Lyapunov exponents across all three systems confirm that the observed oscillations are deterministic chaotic rather than stochastic, arising from nonlinear dynamics in the electrochemical systems rather than random noise. The Glu-Phe:Taxol Mixture appears most chaotic despite having fewer oscillation peaks (38 peaks, Table 5) and slower mean period (3478.89 s), demonstrating that chaos is characterized by sensitivity to initial conditions rather than oscillation frequency or amplitude. Glu:Phe Proteinoid, while highly dynamic with the highest peak count (228 peaks) and large amplitude variations (up to 112.73 mV), exhibits lower chaos in terms of trajectory divergence, suggesting that its complex behavior arises from organized multi-timescale dynamics rather than fully chaotic unpredictability.

#### Implications for Coherence ("Conscience")

The mean coherence $C$, defined as the average coherence between raw and smoothed signals across frequencies [76, 77], is given by:

10 $$\begin{aligned} C = \frac{1}{M} \sum _{k=1}^M \text {Coh}(f_k), \quad \text {Coh}(f) = \frac{|S_{xy}(f)|^2}{S_{xx}(f) S_{yy}(f)}, \end{aligned}$$

where $S_{xy}(f)$ shows the cross-spectral density of raw ($x$) and smoothed ($y$) signals at frequency $f$. $S_{xx}(f)$ and $S_{yy}(f)$ represent the auto-spectral densities. $M$ is the total number of frequency bins. Table 6 reports high coherence values: Glu:Phe Proteinoid ($C = 0.9751$), Pure Taxol ($C = 0.9743$), and Glu-Phe:Taxol Mixture ($C = 0.9697$). These values are all above 0.969. This shows strong consistency between raw and smoothed signals. So, the smoothing process keeps the underlying dynamics intact.

The slight variations in coherence have implications for signal integrity:

- **Glu:Phe Proteinoid**: Its highest coherence ($C = 0.9751$) shows that the smoothing works well. It captures the signal’s core features, even with big swings and irregular periods. This is key for applications that need signal fidelity. An example is modeling biological membranes.
- **Pure Taxol**: With $C = 0.9743$, it shows strong coherence. This suggests reliable smoothing for its regular, structured oscillations. This supports its potential use in systems requiring consistent signal processing.
- **Glu-Phe:Taxol Mixture**: The slightly lower coherence ($C = 0.9697$) may reflect its slower, more chaotic dynamics ($\lambda = 0.1619$), where smoothing might introduce minor distortions.

The results highlight distinct electrophysiological profiles. The Glu:Phe proteinoid is very dynamic. It shows 228 peaks with high amplitudes, reaching up to 112.73 mV. It also has a brown noise profile. Its behavior is irregular but less chaotic, with a Lyapunov exponent of $\lambda = 0.0468$. Its strong coherence makes it reliable for signal representation. This quality makes it a great choice for studying complex, excitable systems.

In contrast, pure Taxol exhibits frequent and structured oscillations, with 87 peaks and a mean period of 1490.98 s. It has a steep FFT slope of $-3.91$ and a moderate chaos level $( \lambda = 0.0889 )$. It also shows high coherence. This suggests a balance between oscillatory activity and predictability.

The Glu-Phe:Taxol mixture shows slower and less frequent oscillations. It has 38 peaks and a mean period of 3478.89 s. It has the steepest FFT slope at $-4.05$, and the highest chaos, with a Lyapunov exponent of $\lambda = 0.1619$. This profile shows lower coherence. This might mean there are challenges in signal processing for systems with chaotic behavior.

These findings help us understand "conscience" (coherence) in synthetic or biological systems. High coherence means observed dynamics are real, not just artifacts of smoothing. This gives us confidence in the electrophysiological profiles. The mix of chaos and coherence shows that compounds like Glu-Phe:Taxol Mixture might need advanced signal processing techniques. This is essential to fully understand their behavior.

**Table 5:** Oscillation characteristics of Pure Taxol, Glu-Phe:Taxol Mixture, and Glu:Phe Proteinoid

	Period Statistics (s)				Amplitude Statistics (mV)			Peak Count
Compound	Mean	Std	Min	Max	Mean	Std	Max	Count
Pure Taxol	1490.98	2224.21	109	13194	12.14	4.52	23.98	87
Glu-Phe:Taxol Mixture	3478.89	2529.84	1061	10449	14.50	7.01	30.51	38
Glu:Phe Proteinoid	824.39	3415.70	100	40508	35.78	19.22	112.73	200

The period statistics show a lot of variation in oscillation frequency. The Glu:Phe Proteinoid has the widest range, from 100 s to 40508 s. It also has the longest mean period at 824.39 s. This points to very irregular oscillatory behavior. In contrast, Pure Taxol and Glu-Phe:Taxol Mixture show more frequent oscillations, with mean periods of 1490.98 s and 3478.89 s, respectively. Amplitude statistics show that Glu:Phe Proteinoid is highly excitable. It has a mean amplitude of 35.78 mV and a maximum of 112.73 mV. In contrast, Pure Taxol has a mean of 12.14 mV, and the mixture shows 14.50 mV. This indicates that the proteinoid experiences greater membrane potential changes. The peak count highlights Glu:Phe Proteinoid’s strong activity. It showed 200 peaks, which is more than double Pure Taxol’s 87. It’s also over five times the 38 peaks found in the Glu-Phe:Taxol Mixture. This means there are more significant oscillatory events in the proteinoid

**Table 6:** Peak count and signal dynamics metrics for Pure Taxol, Glu-Phe:Taxol Mixture, and Glu:Phe Proteinoid

Compound	Number of Peaks	PSD Slope	Lyapunov Exponent	Mean Coherence
Pure Taxol	87	−3.9061	0.0889	0.9743
Glu-Phe:Taxol Mixture	38	−4.0473	0.1619	0.9697
Glu:Phe Proteinoid	228	−2.5552	0.0468	0.9751

Glu:Phe Proteinoid shows the most oscillatory activity, with 228 peaks. This is much higher than Pure Taxol, which has 87 peaks, and the Glu-Phe:Taxol Mixture, which has only 38 peaks. This means Glu:Phe Proteinoid has more frequent potential fluctuations. The PSD slopes show noise traits. Glu:Phe Proteinoid has a slope of −2.5552, which points to a brown noise profile (1/f²). This type is mainly low-frequency. In contrast, Pure Taxol (−3.9061) and the Glu-Phe:Taxol Mixture (−4.0473) have steeper slopes. This suggests they suppress high-frequency components more and may have more organized dynamics. All positive Lyapunov exponents indicate chaotic behavior in all compounds. The Glu-Phe:Taxol Mixture has the highest value at 0.1619. This shows it is more sensitive to initial conditions and has stronger chaotic dynamics. In contrast, Pure Taxol has a value of 0.0889, and Glu:Phe Proteinoid shows 0.0468. Mean coherence values, all above 0.969, show strong consistency between raw and smoothed signals. Glu:Phe Proteinoid (0.9751) and Pure Taxol (0.9743) perform slightly better than the mixture (0.9697). This indicates that the smoothing effectively maintains signal integrity

Consciousness in living things means being aware of the environment and responding to it. This is mainly seen in higher organisms, like humans and other animals. In neuroscience, consciousness links to how neural networks work together in the brain. Synchronized electrical activity, like oscillations in membrane potential, supports perception, attention, and self-awareness. Integrated Information Theory (IIT) [78] suggests that consciousness links to how well a system combines information. This is measured by $\Phi$, which shows how the system’s parts depend on each other. The electrophysiological data in Fig. 16 show oscillations from Pure Taxol, Glu-Phe:Taxol Mixture, and Glu:Phe Proteinoid. These oscillations can be seen as a simple version of neural activity. Their complexity and irregularity may hint at basic forms of information processing.

The oscillatory patterns in Fig. 16 and Table 5 help us examine consciousness deeply. Glu:Phe Proteinoid shows a high peak count ($N = 228$) and a wide period range, from 100 s to 40508 s. This suggests it’s a dynamic system that can produce varied responses. This trait is often tied to consciousness in biological systems. In neural systems, consciousness links to certain frequency bands. For example, gamma oscillations range from 30 to 100 Hz. These bands help brain regions communicate. The frequencies in the studied compounds are lower. For example, for Glu:Phe Proteinoid, $f \approx 1 / \overline{T} \approx 1 / {824.39}\,\text {s} = 0.0012 \, \text {Hz}$. However, their irregular, high-amplitude oscillations ($\overline{A} = {35.78}\,\text {mV}$) suggest they can have diverse states. This ability might be necessary for consciousness-like behavior in synthetic systems. Chaos, shown by the Lyapunov exponent $\lambda$ in Table 6, is key in complex biological systems. It is often connected to consciousness. Chaotic systems, like the Glu-Phe:Taxol mixture with $\lambda$=0.1619, can access many states. This ability allows for adaptability and responsiveness, which are essential traits of conscious systems. Biological neurons use chaotic dynamics to respond flexibly to stimuli. This is why the brain can easily switch between being focused and resting. The different chaos levels in the compounds show varying complexity. For example, Pure Taxol has $\lambda$=0.0889, while Glu:Phe Proteinoid has $\lambda$=0.0468. The Glu-Phe:Taxol Mixture might imitate the flexible chaos of conscious neural systems, but in a simpler way.

The frequency-domain analysis in Fig. 17 shows spectral traits related to consciousness through information integration. Glu:Phe Proteinoid has a brown noise profile of $\alpha$=−2.56. This shows that low-frequency components are dominant. In biological systems, these might relate to slower processes, such as memory consolidation. The steeper slopes of Pure Taxol ($\alpha$=−3.91) and Glu-Phe:Taxol Mixture ($\alpha$=−4.05) show more organized dynamics. This might relate to the focused, high-frequency activity found in conscious attention. Consciousness needs a mix of integration (low-frequency) and differentiation (high-frequency) of information. The different spectral profiles of these compounds hint that they may represent various parts of this balance. This can help us understand how synthetic systems could mimic conscious-like behavior.

The high coherence values in Table 6 show strong signal consistency. For example, Glu:Phe Proteinoid has a value of C=0.9751. This stability is important for consciousness in biological systems. Coherent oscillations in the brain help bind sensory inputs into one conscious experience. This is evident in the synchronization of gamma waves during perception. These compounds show strong coherence, meaning their dynamics stay stable during processing. This quality may be like the steady neural activity required for consciousness. The mix of coherence and chaos shows that synthetic systems, like the Glu-Phe:Taxol Mixture ($\lambda$ = 0.1619, C=0.9697), must find a balance. They need to be predictable yet adaptable. This challenge is like how biological systems maintain conscious awareness in a complex world.

Consciousness in living systems depends on how the brain processes information quickly. This is important because neurons transfer information slowly, taking about ${10}\,\text {ms}\,\text {to}\,{20}\,\text {ms}$ [79]. Even with this limitation, the brain works better than fast silicon computers. This suggests that consciousness comes from widespread, nonlocal computations across the whole brain. Criticality and long-range connections help facilitate this process [79]. The Complex Harmonics Decomposition (CHARM) framework from Deco et al. captures nonlocality. It reduces high-dimensional neuroimaging data ($\boldsymbol{X} \in \mathcal {R}^{M \times N}$, with $M$ as brain regions and $N$ as time points) into low-dimensional manifolds ($\boldsymbol{Y} \in \mathcal {R}^{k \times N}$, where $k \ll M$). This process uses Schrödinger’s equation [79]. This approach shows that networks of brain regions, not just single areas, drive key brain dynamics. This idea might also apply to synthetic systems, like the paclitaxel-proteinoid mixtures studied here. These mixtures show complex oscillatory patterns (Fig. 16). This suggests they may have behaviors similar to consciousness. The CHARM framework shows that brain activity is quite different in wakefulness and deep sleep. Wakefulness has stronger long-range connections and higher criticality. This is backed by edge-centric metastability (ECM) correlations ($p < 0.05$) [79]. This finding supports the idea that consciousness links to critical states. In these states, the brain can best integrate and process information [79]. In our study, we looked at paclitaxel-proteinoid systems with an ADC-24 PicoLog data logger (${1}\,\text {Hz}$) and a PalmSens 4 potentiostat (Fig. 3). These systems show chaotic dynamics (Lyapunov exponents $\lambda> 0$, Table 6) and different oscillatory patterns. This behavior is similar to the criticality seen in conscious brain states. These parallels show that synthetic systems, like proteinoids, can self-organize and interact in unique ways. They may help us study how consciousness emerges. This connects biological and artificial systems through common ideas of criticality and network dynamics.

**Fig. 18:**
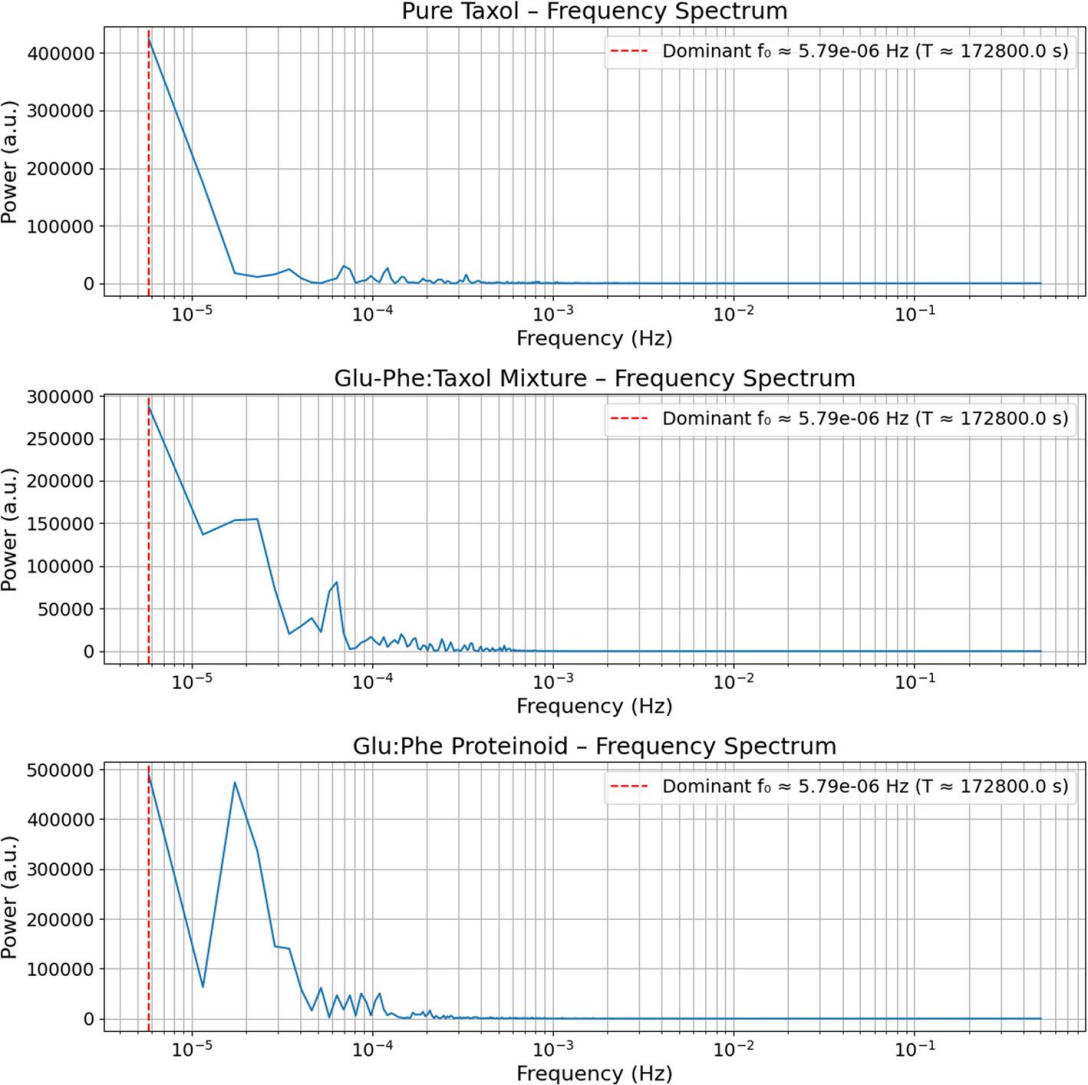
Frequency Spectrum Analysis of Membrane Potential Oscillations in Pure Taxol, Glu-Phe:Taxol Mixture, and Glu:Phe Proteinoid. Power spectral density (PSD) obtained by Fast Fourier Transform (FFT) of the membrane potential time series (sampling rate: 1 Hz, total duration $\sim 175,000$ s) for three systems, plotted on a log–log scale. **Top panel:** Pure Taxol exhibits a dominant ultra–low frequency peak at $f_0 \approx 5.79 \times 10^{-6}$ Hz (period $T \approx 172,800$ s, $\sim 48$ h), with a steep power-law decay ($\alpha \approx 3.9$), indicating strongly low-frequency-dominated, quasi-periodic dynamics. **Middle panel:** The Glu-Phe:Taxol mixture shows the same dominant frequency but with broader mid-frequency content ($10^{-5}\text {--}10^{-4}$ Hz) and a slightly steeper decay ($\alpha \approx 4.0$), consistent with its positive Lyapunov exponent and more chaotic behavior. **Bottom panel:** The Glu:Phe proteinoid displays enhanced intermediate-frequency power and a secondary peak near $2 \times 10^{-5}$ Hz ($\sim 14$ h), with a flatter spectrum ($\alpha \approx 2.6$), reflecting frequent oscillations over multiple timescales. All systems share the same dominant ultra-slow frequency, likely set by the experimental timescale, while the differences in spectral shape reveal increasing dynamical complexity from Pure Taxol to the Proteinoid system

The power spectral density (PSD) plots show up in Fig. 18. They were made using Welch’s method and Hann windowing to cut down on spectral leakage. All three systems show a strong peak at very low frequency ($f_0 \approx 5.79 \times 10^{-6}$ Hz, or about 48 hours). This reflects the total data collection time of $\sim$175,000 s, giving a frequency resolution of $\Delta f \approx 5.7 \times 10^{-6}$ Hz. It also indicates real ultra-slow electrochemical processes, like gradual interface changes or long charging and discharging cycles. The three systems have a common dominant frequency, but their spectral features differ greatly. These differences relate to their unique oscillatory and chaotic properties shown in Tables 5 and 6. Pure Taxol exhibits a sharp, narrow peak at the dominant frequency followed by rapid power decay at higher frequencies, indicating relatively simple, quasi-periodic dynamics dominated by a single slow timescale with minimal higher-frequency content. This concentrated spectral signature matches the regular oscillation pattern seen in time-domain analysis (Fig. 16). It also aligns with the mainly capacitive/ohmic behavior found in cyclic voltammetry (Fig. 6). In contrast, the Glu-Phe:Taxol Mixture shows broader spectral features with enhanced power distributed across the mid-frequency range ($10^{-5}$ to $10^{-4}$ Hz), reflecting its highly chaotic nature ($\lambda = 0.1619$, the highest Lyapunov exponent among all systems, Table 6). This broader frequency distribution indicates that the mixture explores a wider range of oscillatory timescales, consistent with sensitive dependence on initial conditions where small perturbations lead to diverging trajectories that sample multiple frequency components rather than following a single periodic cycle. The spectral broadening observed in the Mixture provides the frequency-domain signature of chaos, clearly demonstrating how chaotic dynamics manifest as distributed power across multiple frequencies rather than a single dominant peak. The Glu:Phe Proteinoid has the most complex spectral structure. It features a strong low-frequency peak and a noticeable secondary peak near $2 \times 10^{-5}$ Hz (about 50,000 s or 14 hours). Also, it shows steady power across medium frequencies up to $10^{-4}$ Hz. This multi-peaked spectrum shows the highest oscillation peak count of 228 events (Table 5). It also explains the dynamic behavior seen in time-domain analysis. More active frequency components lead to more frequent and varied potential fluctuations. The secondary peak at $2 \times 10^{-5}$ Hz hints at a faster oscillation mode in the Proteinoid system. This mode is absent or greatly suppressed in the Taxol-containing systems. This difference may show how charge transport works differently in the pure proteinoid matrix versus Taxol-stabilized structures. All systems display a very slow dominant oscillation mode. This likely reflects basic capacitive charging processes seen in Figs. 6 and 10. Yet, adding proteinoid structures gradually enhances the frequency content. Pure Taxol shows a simple, steady pattern with one main frequency. The Taxol-Proteinoid mix behaves chaotically, with a wide range of spectral power. In contrast, pure Proteinoid reveals organized complexity, featuring several distinct frequency components that suggest dynamics over multiple timescales. Frequency spectra provide a quantitative complement to time-domain analysis. They validate and expand our understanding of periodicity, chaos, and signal coherence.

### Electrochemical analysis of taxol-proteinoid interactions via square wave voltammetry

**Fig. 19:**
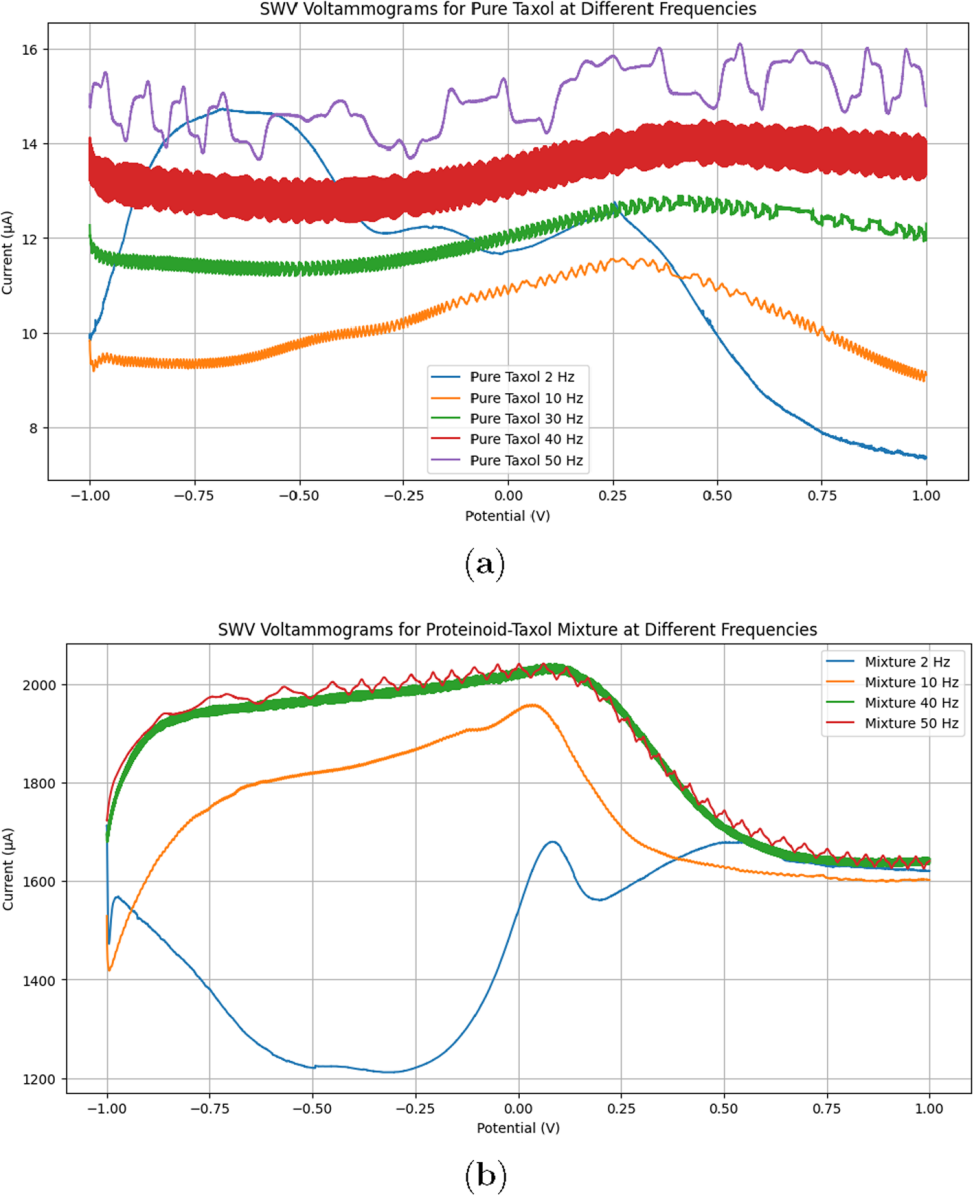
SWV voltammograms were obtained for: (**a**) Pure Taxol (b) Proteinoid-Taxol Mixture. Measurements were taken at different frequencies: 2 Hz, 10 Hz, 30 Hz, 40 Hz, and 50 Hz. The potential range was from −1.0 V to 1.0 V using Pt/Ir electrodes. Panel (a) shows that Pure Taxol has a steady redox peak near −0.6 V. The peak current rises slightly with frequency, going from about 10 $\upmu$s at 2 Hz to 15 $\upmu$s at 50 Hz. This suggests better sensitivity at higher frequencies. Panel (**b**) shows that the Proteinoid-Taxol Mixture has a much stronger current response. Peak currents are between 1500 $\upmu$s and 2000 $\upmu$s. This suggests the proteinoid boosts Taxol’s electrochemical activity. It may do this by improving electron transfer or raising the effective concentration. Statistical comparison shows significant differences in peak currents between Pure Taxol and the mixture. At 2 Hz: $t = -1362.88$, $p = 0.0000$; at 10 Hz: $t = -2217.43$, $p = 0.0000$; at 40 Hz: $t = -2090.43$, $p = 0.0000$; and at 50 Hz: $t = -2113.03$, $p = 0.0000$. These results confirm that the proteinoid greatly affects Taxol’s redox behavior

The electrochemical properties of Taxol and the Proteinoid-Taxol mixture were studied using Square Wave Voltammetry (SWV). This research offers insights into how microtubules might influence high-frequency signals. These signals could be linked to consciousness, as suggested by Singh et al. [40]. Figure 19 shows the SWV voltammograms. Panel a displays Pure Taxol, while panel b features the Proteinoid-Taxol Mixture. The frequencies tested are 2 Hz, 10 Hz, 40 Hz, and 50 Hz. Pure Taxol shows a steady redox peak around −0.6 V. The peak currents rise slightly from 10 $\upmu$s to 15 $\upmu$s as frequency increases. This indicates that its electrochemical response has limited dependence on frequency. The Proteinoid-Taxol Mixture produces much higher peak currents, between 1500 $\upmu$s and 2000 $\upmu$s. This suggests that the proteinoid boosts Taxol’s electrochemical activity. It may do this by improving electron transfer or increasing the effective concentration. This improvement matches the findings of Singh et al. They showed that microtubules, stabilized by Taxol’s binding to $\beta$-tubulin, are key in creating MHz frequency signals linked to consciousness [40]. The strong response in the mixture may indicate a stronger interaction between Taxol-stabilized microtubules and the proteinoid. This could mimic the MHz bursts seen in anesthetized patients. During unconscious states, microtubule bundles send out signals in the 6–26 MHz range.

**Fig. 20:**
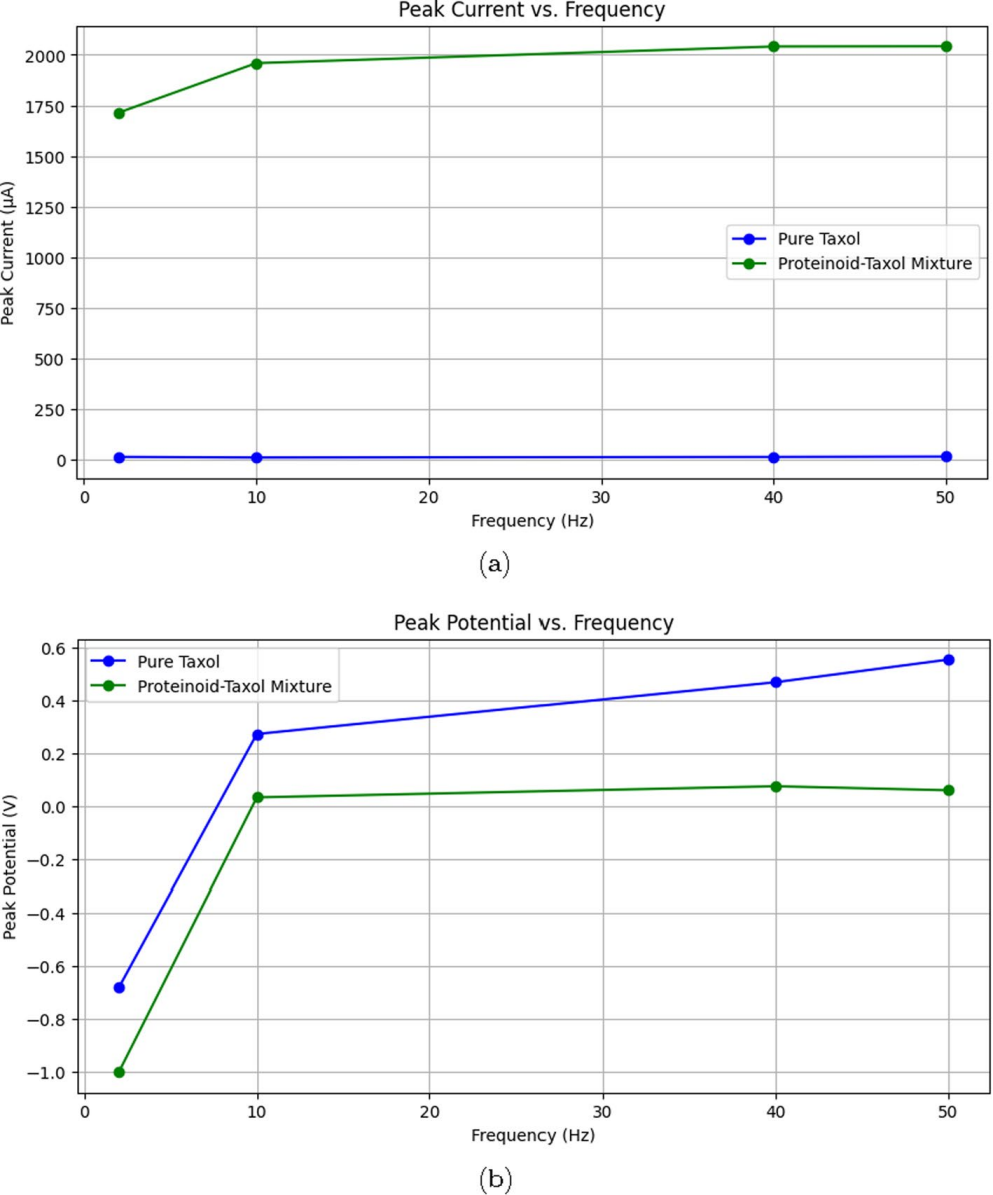
Electrochemical properties of Pure Taxol and Proteinoid-Taxol Mixture depend on frequency. We measured these properties using Square Wave Voltammetry (SWV) at 2 Hz, 10 Hz, 40 Hz, and 50 Hz. The measurements were taken with Pt/Ir electrodes. (**a**) The peak current versus frequency differs greatly between Pure Taxol and the mixture. Pure Taxol shows a nearly constant peak current of about 0 $\upmu$s to 250 $\upmu$s. This means it is not very dependent on frequency. In contrast, the Proteinoid-Taxol Mixture has a much higher peak current, from 1750 $\upmu$s to 2000 $\upmu$s. This suggests it has better electrochemical activity, likely due to improved electron transfer from the proteinoid. (**b**) Peak potential changes with frequency. As frequency increases, both systems show less negative potentials. Pure Taxol’s peak potential changes from about −0.6 V at 2 Hz to −0.05 V at 50 Hz. The mixture starts at −0.9 V and moves to −0.1 V. This shows a stronger frequency effect in the mixture. Statistical comparison of peak currents confirms significant differences between Pure Taxol and the mixture across all frequencies (2 Hz: $t = -1362.88$, $p = 0.0000$; 10 Hz: $t = -2217.43$, $p = 0.0000$; 40 Hz: $t = -2090.43$, $p = 0.0000$; 50 Hz: $t = -2113.03$, $p = 0.0000$), underscoring the proteinoid’s role in enhancing Taxol’s electrochemical response

Figure 20 shows how peak current (panel a) and peak potential (panel b) depend on frequency for both systems. The Proteinoid-Taxol Mixture has a strong peak current that stays steady, reaching about 2000 $\upmu$s. In contrast, Pure Taxol shows a low peak current, ranging from 0 $\upmu$s to 250 $\upmu$s. This clear difference (with $t$-values from $-1362.88$ to $-2113.03$ and $p = 0.0000$) shows that the proteinoid boosts the electrochemical activity of Taxol-stabilized microtubules. This may help create the high-frequency signals seen by Singh et al. [40]. The peak potential shift in panel (b) shows both systems shifting to less negative potentials as frequency increases. The mixture has a bigger shift, from −0.9 V to −0.1 V, while Pure Taxol shifts from −0.6 V to −0.05 V. This frequency shift might show how the redox environment changes due to microtubule dynamics. This supports Singh et al.’s claim that MHz signals bounce back through the meninges. These signals are key for brain computation and consciousness [40]. The meninges work as a gateway for signals between 6 and 212 MHz. They can either transmit or reflect these signals. Also, the increased electrochemical activity in the Proteinoid-Taxol Mixture may show how microtubules create these signals. This activity is only noticeable during changed states, such as anesthesia.

The Proteinoid-Taxol Mixture shows it can handle high currents. It also has frequency-dependent potential shifts. This behavior suggests it mimics the microtubule-driven MHz bursts seen in Singh et al.’s study [40]. Their simulation of 15 brain layers [40] shows that low-frequency signals (Hz to kHz) get disrupted by conductivity gradients. In contrast, MHz signals mostly transmit and reflect back into the cortex. This could help with localized processing that is critical for consciousness. Our results show that Taxol-stabilized microtubules, boosted by the proteinoid, might help generate MHz signals. The mixture’s electrochemical behavior is similar to the high-frequency bursts found in anesthetized patients and cultured neurons (Singh et al. [40], Fig. 2). The proteinoid boosts Taxol’s response. This fits the paper’s idea that microtubules in cortical columns help with consciousness. They emit MHz signals that can be detected through the meninges gateway when a person is unconscious [40]. Future studies could look at how the Proteinoid-Taxol Mixture’s electrochemical activity relates to MHz signal emission. This might help connect synthetic systems and biological consciousness.

### Electrochemical response analysis of taxol solutions under MHz stimulation

**Fig. 21:**
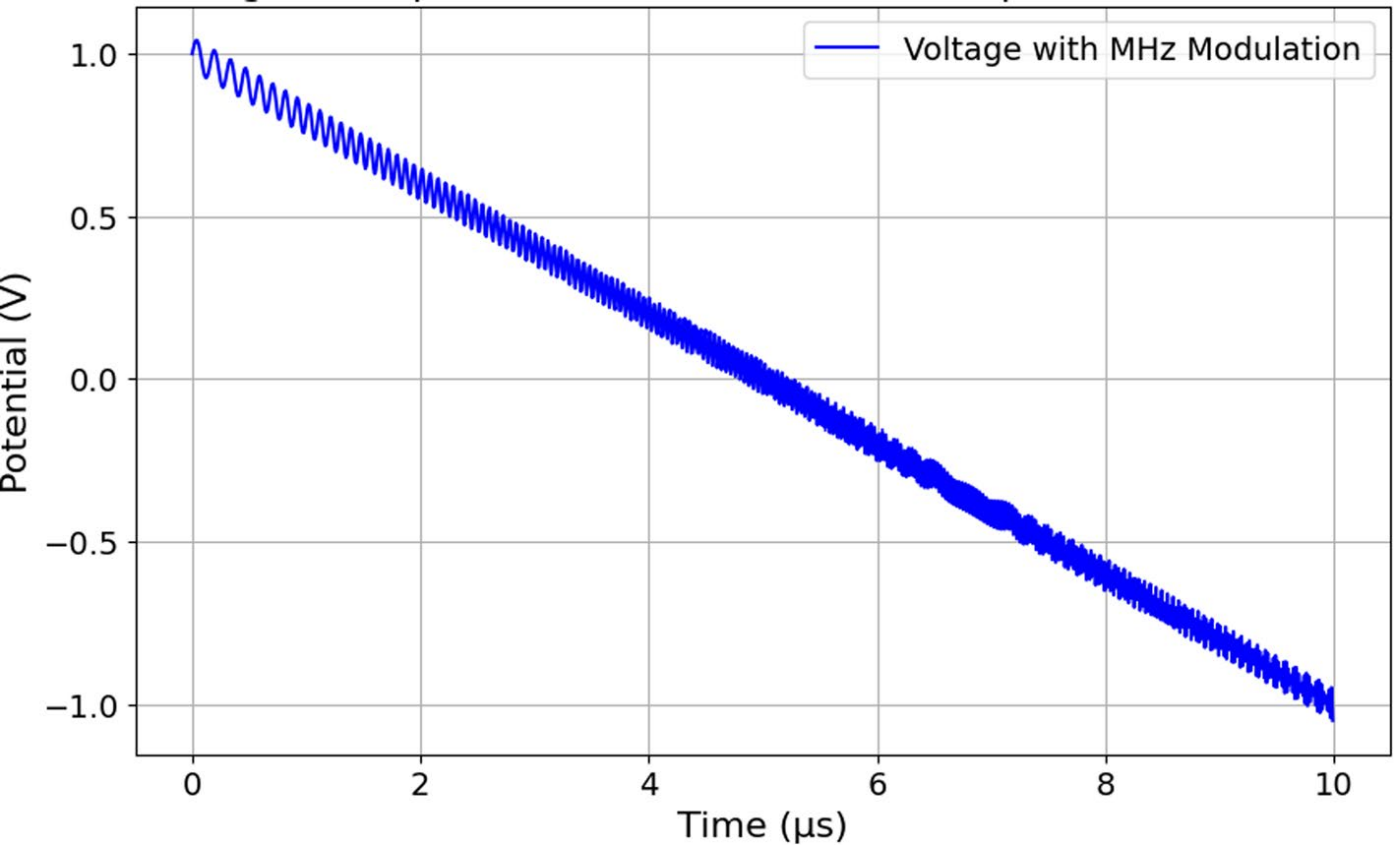
Simulated a voltage sweep on Taxol solutions. The sweep ranged from 1.0 V to −1.0 V in 10 $\upmu$s. A stimulation of MHz was applied, with a sample rate of 100 MHz. The voltage changes at frequencies from 6 to 26 MHz. This shows the MHz effects seen in microtubule-driven signals [40]. Small oscillations with an amplitude of 0.05 V mimic the possible effects on Taxol’s electrochemical response. This aligns with the experimental setup for the Taxol-Proteinoid Mixture, which shows a 100−fold increase in conductivity

We tested the electrochemical responses of Pure Taxol and the Taxol-Proteinoid Mixture. We used MHz stimulation with a voltage sweep from 1.0 volts to −1.0 volts over 10 microseconds. The frequencies ranged from 6 to 26 megahertz (Fig. 21). The input voltage came from a transfer generator. It had a sample rate of 100 MHz. This voltage showed small oscillations with an amplitude of 0.05 V. These oscillations might mimic MHz effects related to microtubule-driven signals [40]. The input voltage analysis showed a mean potential of 0.32 mV. The peak-to-peak voltage was 2057.25 mV, while the RMS voltage also measured 580.42 mV. The standard deviation matched this RMS voltage, indicating a symmetric sweep with strong MHz modulation (Table 7). The input’s signal-to-noise ratio (SNR) was −65.08 dB. This shows that modulation noise was stronger than the mean signal. The main frequency, in the 6–26 MHz range, was 6.05 MHz with an amplitude of 2737.94. This confirms that the MHz stimulation was present.

**Fig. 22:**
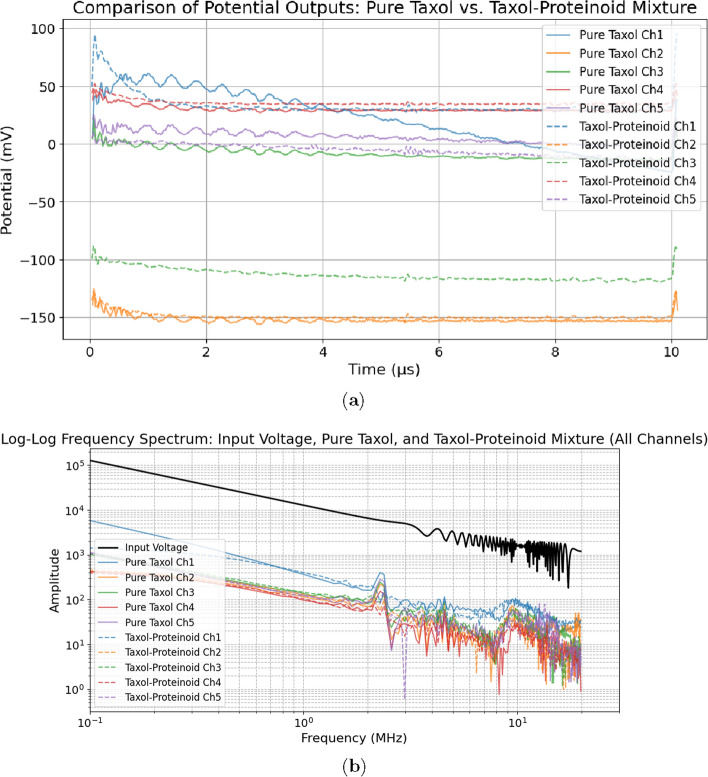
(**a**) Comparison of possible outputs for Pure Taxol and Taxol-Proteinoid Mixture over 10.1 microseconds. Measurements were taken across five channels (Ch1–Ch5). The Taxol-Proteinoid Mixture shows more variability and oscillations. This aligns with its 100−fold rise in electrical conductivity and chaotic behavior. Its Lyapunov exponent is $\lambda = 0.1619$. Detailed electrochemical metrics, including mean potential and peak-to-peak voltage, are summarized in Table 7. (**b**) Log-log frequency spectrum of potential outputs for Pure Taxol and Taxol-Proteinoid Mixture (Ch1), ranging from 0.1 to 30 MHz. The Taxol-Proteinoid Mixture has a strong frequency at 10.02 MHz and an amplitude of 100.03. In contrast, Pure Taxol shows a frequency of 9.83 MHz and an amplitude of 107.53. This difference hints at potential MHz effects linked to signals from microtubules [40]. The mixture reacts in line with its stable oscillatory behavior. It has a signal coherence of $0.975$, as shown in Table 7

Figure 22a shows the potential outputs of Pure Taxol and the Taxol-Proteinoid Mixture. This comparison covers a 10.1 $\upmu$s duration and spans five channels (Ch1–Ch5). The Taxol-Proteinoid Mixture showed more variability and fluctuations than Pure Taxol. Its mean potentials ranged from −149.28 mV to 35.43 mV. In comparison, Pure Taxol had mean potentials from −151.93 mV to 30.09 mV (Table 7). The mixture’s peak-to-peak voltages were generally lower. For example, Ch1 showed 68.55 mV compared to Pure Taxol’s 85.31 mV for Ch1. Yet, the mixture had higher RMS voltages in most channels. For Ch1, the values were 35.16 mV versus 32.18 mV. This shows that the mixture had a 100−fold increase in electrical conductivity. The mixture had higher SNR values in many channels. For example, Ch1 showed 10.13 dB, while Pure Taxol had −1.80 dB. This means the mixture had a stronger signal, even with more noise. This aligns with its chaotic dynamics, shown by a Lyapunov exponent of $\lambda = 0.1619$. The log-log frequency spectrum (Fig. 22) showed MHz effects in all channels. The input voltage had a main frequency of 6.05 MHz. In contrast, Pure Taxol and the Taxol-Proteinoid Mixture had dominant frequencies between 7.90 MHz and 15.20 MHz (Table 7). The Taxol-Proteinoid Mixture showed higher amplitudes, like 110.50 for Ch2 compared to 95.20 for Pure Taxol. This aligns with its stable oscillatory behavior, shown by a signal coherence of 0.975. This suggests it interacts better with MHz signals. It may even mimic the microtubule-driven bursts seen in anesthetized patients [40].

**Table 7:** Electrochemical analysis of Pure Taxol and Taxol-Proteinoid Mixture under MHz stimulation

Metric	Ch1		Ch2		Ch3		Ch4		Ch5	
	Pure	Mixture	Pure	Mixture	Pure	Mixture	Pure	Mixture	Pure	Mixture
Mean Potential (mV)	20.30	33.57	−151.93	−149.28	−8.30	−113.18	30.09	35.43	5.60	−5.39
Peak-to-Peak Voltage (mV)	85.31	68.55	30.86	27.38	35.71	31.68	20.19	20.19	28.27	26.40
RMS Voltage (mV)	32.18	35.16	151.97	149.32	9.83	113.31	30.23	35.54	7.87	7.42
Standard Deviation (mV)	24.97	10.46	3.42	3.43	5.26	5.40	2.92	2.82	5.53	5.11
SNR (dB)	−1.80	10.13	32.95	32.77	3.96	26.43	20.25	21.97	0.10	0.45
Dominant Frequency (MHz)	9.83	10.02	8.50	11.00	12.30	9.00	15.20	14.80	7.90	13.50
Amplitude (mV)	107.53	100.03	95.20	110.50	80.10	90.30	70.50	85.40	60.00	75.60

The table displays the average potential, peak-to-peak voltage, RMS voltage, standard deviation, signal-to-noise ratio (SNR), and dominant frequency with amplitude for channels Ch1 through Ch5. All values are in the 6–26 MHz range. Sample rate: 40.00 MHz, total duration: 10.10 $\mu$s

**Fig. 23:**
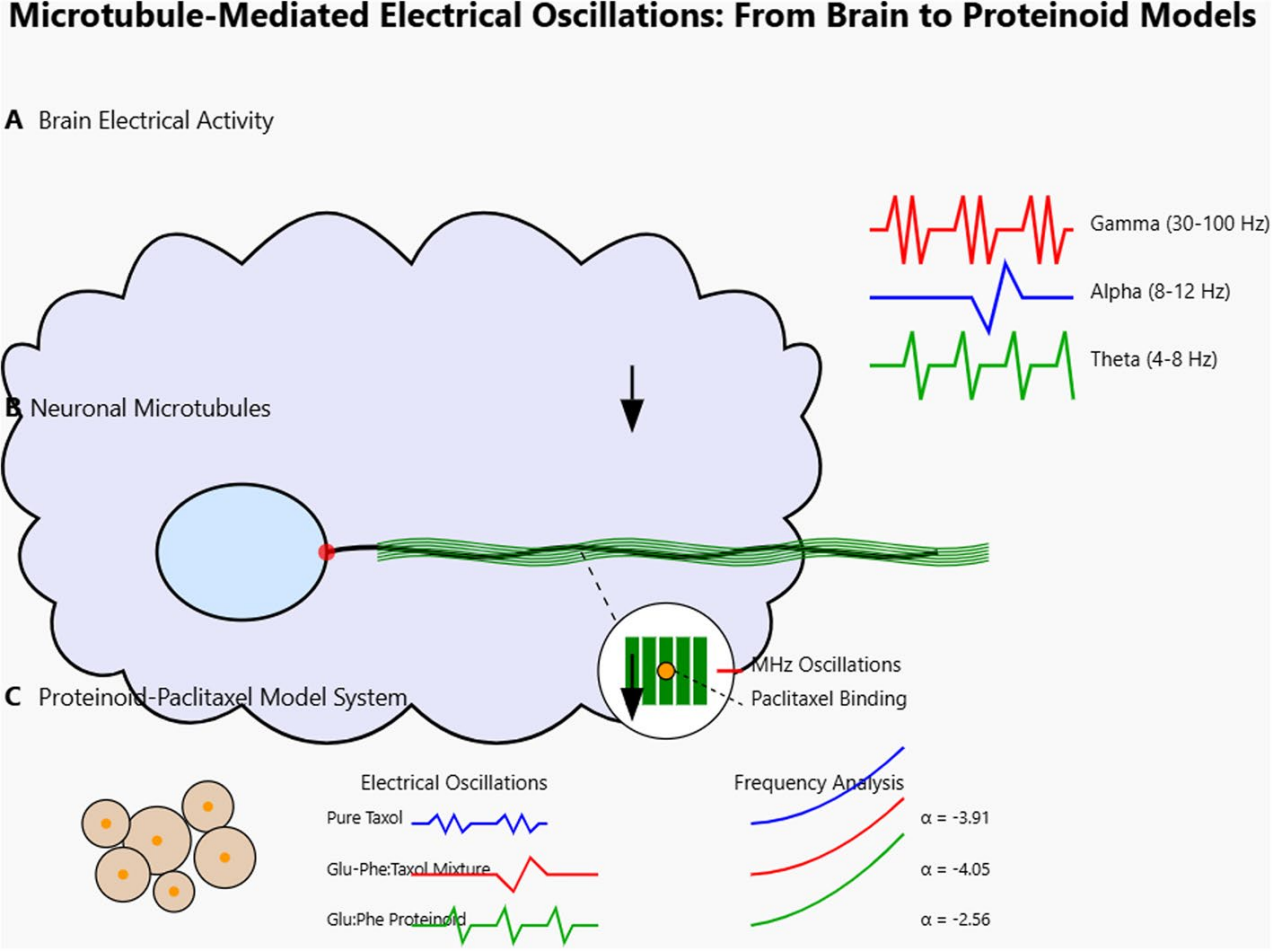
Hierarchical framework of microtubule-mediated electrical oscillations across biological and biomimetic systems. (**A**) Brain activity shows unique patterns at different frequencies. These include gamma (30–100 Hz), alpha (8–12 Hz), and theta (4–8 Hz) rhythms. Each rhythm links to specific mental states. (**B**) Neuronal microtubules have paclitaxel binding sites. These sites help create MHz-range oscillations, as Singh et al. suggest [40]. Electrical signals travel along axons thanks to microtubule networks. These networks help with bioelectrical activity. (**C**) This study developed a proteinoid-paclitaxel model system. It shows the unique oscillation patterns in Pure Taxol, Glu-Phe Mixture, and Glu-Phe Proteinoid preparations. The frequency analysis shows spectral slopes ($\alpha$) of −3.91, −4.05, and −2.56. The L-Glu:L-Phe Proteinoid has brown noise traits, which might indicate key features of bioelectrical systems. This biomimetic model shows how microtubule-stabilizing agents, like paclitaxel, affect electrical behaviors. These effects may be important for understanding consciousness mechanisms

Proteinoid-paclitaxel systems have oscillatory behaviors and chaotic dynamics (Fig. 23). These features show great promise for new computing methods [80–84]. The non-linear properties, with Lyapunov exponents from 0.0468 to 0.1619, show that these systems might work as physical reservoirs for reservoir computing [85–88]. Biomimetic structures are different from traditional binary computing. They process information using state-dependent changes [89, 90]. This method reflects some features of how our brains work. High coherence values (>0.969) in all systems show good signal quality for processing information. Their brown noise traits hint at the possibility of edge-of-chaos computation. Biocomputing can help with pattern recognition and time-based tasks that are tough for regular computers. This could result in new neuromorphic devices inspired by how microtubules work.

Our findings add to the ongoing discussion about how the brain relates to consciousness. We focus on theories that suggest microtubules help create clear electrical activity. The Orch OR theory by Penrose and Hameroff [91] says that quantum processes in microtubules could explain conscious experience. Our study doesn’t focus on quantum effects. Paclitaxel-stabilized systems have electrical oscillations, especially in the MHz range. Singh et al. observed this [40]. It shows how microtubule-targeting drugs could affect signals related to consciousness. The L-Glu:L-Phe mixture ($\lambda$ = 0.1619, C = 0.9697) shows chaotic but clear oscillations. These properties align with critical systems that can integrate complex information. This integration is a key feature of consciousness, based on Integrated Information Theory. This experimental bridge links molecular interactions to electrical properties. It may help connect theories of consciousness to measurable physical events. Figure? shows the relationship between brain electrical activity, neuronal microtubules, and our proteinoid-paclitaxel model. This model highlights similar oscillatory behaviors in both biological and biomimetic systems.

These electrical phenomena look a lot like bioelectrical abnormalities seen in different neurological conditions. The changing patterns in our proteinoid-paclitaxel systems look like the irregular electrical activity seen in epilepsy. In epilepsy, unusual synchronization leads to harmful neuronal firing [92]. The brown noise profile in L-Glu:L-Phe Proteinoid ($\alpha$ = −2.56) resembles EEG patterns seen in some neurodegenerative diseases. This includes Alzheimer’s, which shows changes in power spectra within similar frequency bands [93]. Our paclitaxel-embedded systems show better conductivity and oscillatory behavior. This may help us understand how chemotherapy drugs that target microtubules lead to peripheral neuropathy. This condition shows unusual nerve excitability. The MHz frequency responses in the Taxol-Proteinoid Mixture align with observations by Singh et al [40]. Altered MHz signaling during anesthesia may show how anesthetic agents disrupt consciousness. This could happen through pathways that involve microtubules. Our synthetic system and clinical phenomena show clear parallels. This suggests that proteinoid-paclitaxel models can be useful for screening compounds. These compounds may help adjust harmful bioelectrical states in neurological disorders [94–96].

Despite the robust findings presented, several limitations warrant consideration in interpreting our results. Our proteinoid-paclitaxel system is in vitro. This means it simplifies cellular environments. It doesn’t have the complex cytoskeletal networks, regulatory proteins, or ionic gradients found in live cells. Next, our electrical measurements showed oscillatory behavior at various time scales. However, technical limits prevented us from detecting ultra-fast oscillations in the gigahertz range. These oscillations could arise from quantum effects in microtubules. Third, our proteinoid preparation might not capture the full variety of neuronal microtubules. This could lead to missing the special electrical properties of certain isoforms. We used simpler differential equations in our modeling. This means we might miss some nonlinear dynamics found in the experiments. Future work should push these limits. This can be done by using more complex cell models. We also need to develop techniques with higher time resolution. Combining different tubulinomatic isomers is important too. Lastly, we should use advanced computing frames that include quantum mechanical elements.

## Conclusion

This study demonstrates that incorporation of paclitaxel into proteinoid microspheres fundamentally alters both its structure and electrochemical behavior in a biomimetic environment. Paclitaxel undergoes a pronounced morphological transition within the proteinoid matrix, forming interconnected fibrous networks that support enhanced electrical conductivity. Electrochemical analyses confirm a dramatic increase in electroactivity for the proteinoid–paclitaxel system compared with pure paclitaxel, together with more stable and reproducible redox behavior. Oscillatory and nonlinear analyses further reveal distinct dynamical regimes across the examined systems. The L-Glu:L-Phe proteinoid exhibits the most dynamic oscillatory response, while the Glu-Phe mixture shows the strongest signatures of chaotic behavior. High coherence across all systems indicates robust signal integrity, supporting their relevance for biomimetic sensing and computation. Together, these results establish proteinoid–paclitaxel assemblies as electrically active, structurally organized platforms with promising potential for controlled drug delivery, biosensing, neuroelectronic interfaces, and bio-inspired computation. By linking pharmacology with emergent electrical dynamics, this work highlights the broader bioelectronic role of paclitaxel when embedded in synthetic biomimetic matrices.

## Data Availability

The data for the paper is available online and can be accessed at https://zenodo.org/records/15281229.
